# WhiskEras: A New Algorithm for Accurate Whisker Tracking

**DOI:** 10.3389/fncel.2020.588445

**Published:** 2020-11-17

**Authors:** Jan-Harm L. F. Betting, Vincenzo Romano, Zaid Al-Ars, Laurens W. J. Bosman, Christos Strydis, Chris I. De Zeeuw

**Affiliations:** ^1^Department of Neuroscience, Erasmus MC, Rotterdam, Netherlands; ^2^Department of Quantum & Computer Engineering, Delft University of Technology, Delft, Netherlands; ^3^Netherlands Institute for Neuroscience, Royal Academy of Arts and Sciences, Amsterdam, Netherlands

**Keywords:** sensorimotor integration, whiskers, object tracking, algorithm, cerebellum, Purkinje cell, mouse, machine learning

## Abstract

Rodents engage in active touch using their facial whiskers: they explore their environment by making rapid back-and-forth movements. The fast nature of whisker movements, during which whiskers often cross each other, makes it notoriously difficult to track individual whiskers of the intact whisker field. We present here a novel algorithm, WhiskEras, for tracking of whisker movements in high-speed videos of untrimmed mice, without requiring labeled data. WhiskEras consists of a pipeline of image-processing steps: first, the points that form the whisker centerlines are detected with sub-pixel accuracy. Then, these points are clustered in order to distinguish individual whiskers. Subsequently, the whiskers are parameterized so that a single whisker can be described by four parameters. The last step consists of tracking individual whiskers over time. We describe that WhiskEras performs better than other whisker-tracking algorithms on several metrics. On our four video segments, WhiskEras detected more whiskers per frame than the Biotact Whisker Tracking Tool. The signal-to-noise ratio of the output of WhiskEras was higher than that of Janelia Whisk. As a result, the correlation between reflexive whisker movements and cerebellar Purkinje cell activity appeared to be stronger than previously found using other tracking algorithms. We conclude that WhiskEras facilitates the study of sensorimotor integration by markedly improving the accuracy of whisker tracking in untrimmed mice.

## 1. Introduction

Most mammals, with humans being one of the very few exceptions, use whiskers to orient themselves. Typically, whiskers are found on the cheek and around the mouth and assist with feeding, but a number of species have evolved highly specialized functions of their whiskers (Ahl, 1986; Sokolov and Kulikov, 1987; Bosman et al., 2011). Herbivorous manatees, dwelling in tropical waters, can orient themselves by detecting minute water flows around objects with their whiskers (Sarko et al., 2007; Gaspard et al., 2013, 2017), while carnivorous pinnipeds use their whiskers to detect vibrations in the water caused by prey fish tens of meters away (Dehnhardt et al., 2001; Schulte-Pelkum et al., 2007). Also aerial squirrels have unusually large whiskers that they use during gliding (Ahl, 1987), but the most extensively studied whisker system is that of small rodents like mice and rats: behavioral as well as anatomical aspects of the rodent whisker system have made it a popular model system to study neurodevelopment and sensorimotor integration as well as to an inspiration for robotics (Vincent, 1913; Welker, 1964; Woolsey et al., 1975; Carvell and Simons, 1990; Fox, 1992; Svoboda et al., 1997; Brecht and Sakmann, 2002; Brecht, 2007; Diamond et al., 2008; Prescott et al., 2009; Bosman et al., 2011; Hartmann, 2011; Petersen, 2019).

Mice, rats and some other small rodents and insectivores can move their large facial whiskers rhythmically with frequencies of 5–20 Hz, occasionally up to around 30 Hz, a speed that is rather unique in mammals (Berg and Kleinfeld, 2003; Knutsen et al., 2006; Munz et al., 2010; Rahmati et al., 2014). In doing so, they can actively explore their environment and establish the shapes and textures of the objects around them (Rodgers et al., 2020). Neural interpretation of such active touch is computationally demanding and consequently a large part of the rodent brain is related to the whisker system (Kleinfeld et al., 1999; Bosman et al., 2011). Whisker input can be used to trigger associative learning with key roles for the somatosensory and (pre)motor cortex, and the cerebellum (Leal-Campanario et al., 2006; Troncoso et al., 2007; O'Connor et al., 2010b; Rahmati et al., 2014; Gao et al., 2018). From an experimental point of view, understanding the behavioral context and thus (also) the whisker movements is crucial for the interpretation of neural activity in relation to the whisker system. Neural activity is context-dependent, and sensory gating is attenuated during self-movement (Fanselow and Nicolelis, 1999; Lee et al., 2008; Chakrabarti and Schwarz, 2018). Given the intrinsic complexity of the whisker system, with a prominent somatotopic representation of individual whiskers in the trigeminal nuclei, the thalamus and the primary somatosensory cortex (Woolsey et al., 1975; Bosman et al., 2011), as well as the ability of mice to move whiskers individually (Dörfl, 1985; Simony et al., 2010), tracking of individual whiskers is desirable.

The fast speed of the many whiskers organized in different rows severely complicates accurate tracking of their movement. In the past, several approaches have been taken to address this problem. Many of these involve the clipping of all but one or a few whiskers (Bermejo et al., 2002; Knutsen et al., 2005, 2008; Voigts et al., 2008; O'Connor et al., 2010a; Clack et al., 2012; Dorschner et al., 2016; Nashaat et al., 2017; Sehara et al., 2019; Petersen et al., 2020). Although animals are astonishingly good at obtaining sensory input from a single spared whisker (O'Connor et al., 2010a), whisker clipping is a rather unsatisfactory method from a behavioral point of view (Pluta et al., 2017). Alternatively, whiskers can be labeled with small markers (Herfst and Brecht, 2008; Roy et al., 2011). However, even small markers are heavy in comparison to the mass of the whisker itself and therefore affect its movement. As far as we are aware, only the BIOTACT Whisker Tracking Tool (BWTT) aims at tracking unclipped and unlabeled whiskers (Perkon et al., 2011). BWTT is, however, not designed to follow the trajectories of individual whiskers within the whisker field. To overcome this limitation, we have previously introduced *post-hoc* processing of data generated with BWTT (Rahmati et al., 2014; Ma et al., 2017; Romano et al., 2018). *Post-hoc* processing of the BWTT data in order to follow individual whiskers turned out to be relatively inaccurate in untrimmed mice. BWTT only determines the angle and position of a whisker shaft on a specific distance from the snout, which, given the fact that whiskers tend to fan out, is not always a good approximation of the actual angle and position. Furthermore, BWTT cannot detect whisker shafts with subpixel accuracy, which complicates the distinction and tracking of individual whiskers over time. These limitations motivated us to develop a new whisker tracking tool, WhiskEras, which we present here.

From a computational perspective, processing videos with frequencies of 1,000 Hz in a reasonable amount of time is challenging. Recently, we described that the computational load of BWTT, which was implemented in MATLAB, grows exponentially with the number of detected whisker points (Ma et al., 2017). By porting the BWTT algorithm to OpenMP, making use of parallelism, we managed to achieve a speedup of 4,500x (Ma et al., 2017; Romano et al., 2020). Similar to BWTT, on which it is partially based, WhiskEras was implemented in MATLAB, but - in contrast to the original version of BWTT - WhiskEras makes use of the multicore CPU as well as a GPU to achieve reasonable processing times.

In contrast to BWTT, but similar to Janelia Whisk (Clack et al., 2012) and older trackers (Voigts et al., 2008), WhiskEras takes the shape of the whiskers into account. WhiskEras uses shape parameters to follow individual whiskers within the unclipped, unlabeled whisker field. Although also deep learning approaches provide promising results (Mathis et al., 2018), we decided to use a computer-vision approach. We show here in direct comparisons that under our recording conditions, the accuracy of WhiskEras outperforms that of Whisk and BWTT.

## 2. Materials and Methods

### 2.1. Whisker Videos and Electrophysiology

For the development, testing and validation of WhiskEras, we used high-speed videos of head-fixed mice that were made for a previously published study (Romano et al., 2018). The details of the recordings are described in that paper. Briefly, adult C57Bl6/J mice received a magnetic pedestal with which they could be head-fixed in the recording setup. After habituation, video recordings of the whiskers of the right side of the head were made (750–1,000 Hz full-frame rate, 480 × 512 pixels using a red LED panel as backlight). Extracellular single-unit recordings of Purkinje cells in the ipsilateral cerebellar lobules crus 1 and crus 2 were made using quartz-platinum electrodes (Thomas Recording, Giessen, Germany), digitized and stored at 24 kHz after 1–6,000 Hz filtering (RZ2 multi-channel workstation, Tucker-Davis Technologies, Alachua, FL, USA). All recordings were made in awake mice in the absence of whisker clipping or marking. Every video originated from another mouse.

### 2.2. Pixel-Level Processing

**Figure 1:**
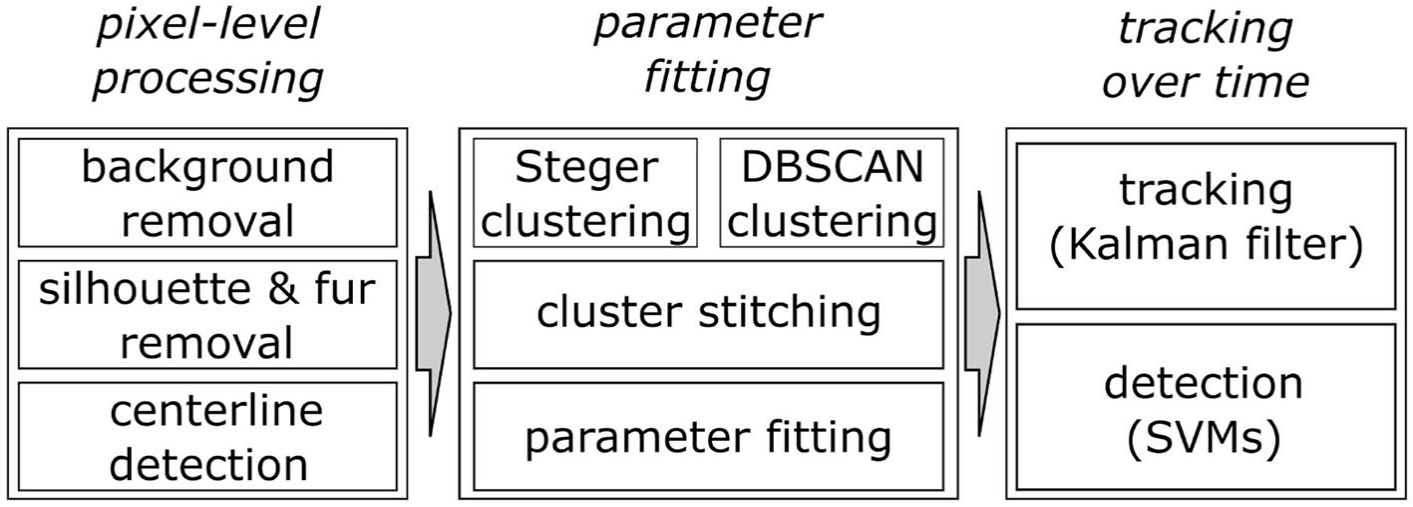
Overview of the three main processing steps of the WhiskEras algorithm and their sub-steps.

**Figure 2:**
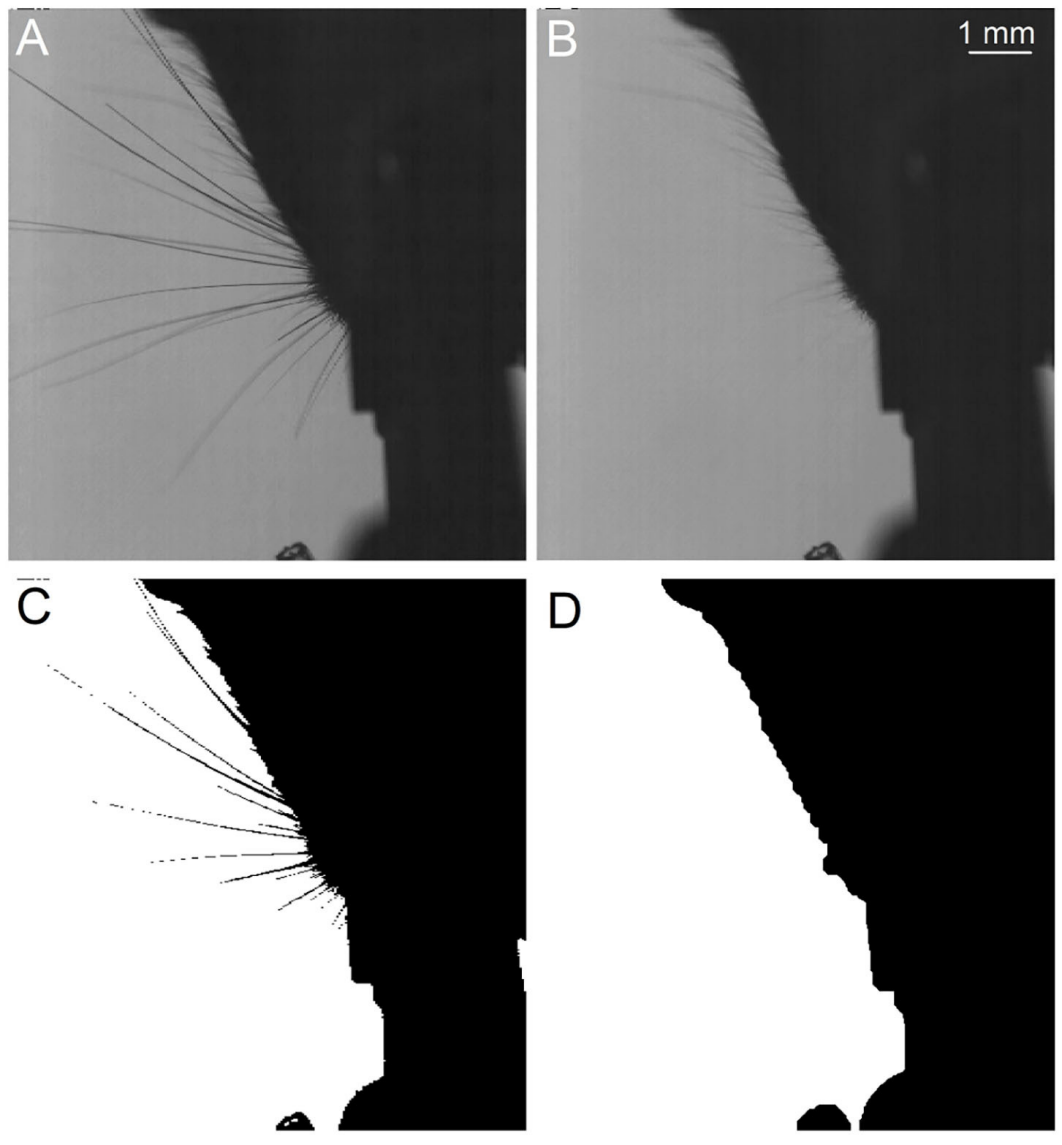
Procedure to obtain the background and the snout silhoutte, which will both be subtracted from the frames. **(A)** Original frame taken from above. **(B)** Extracted background. **(C)** Binarized image, still showing the whiskers. **(D)** Snout silhouette after removing the whiskers from the binarized image.

**Figure 3:**
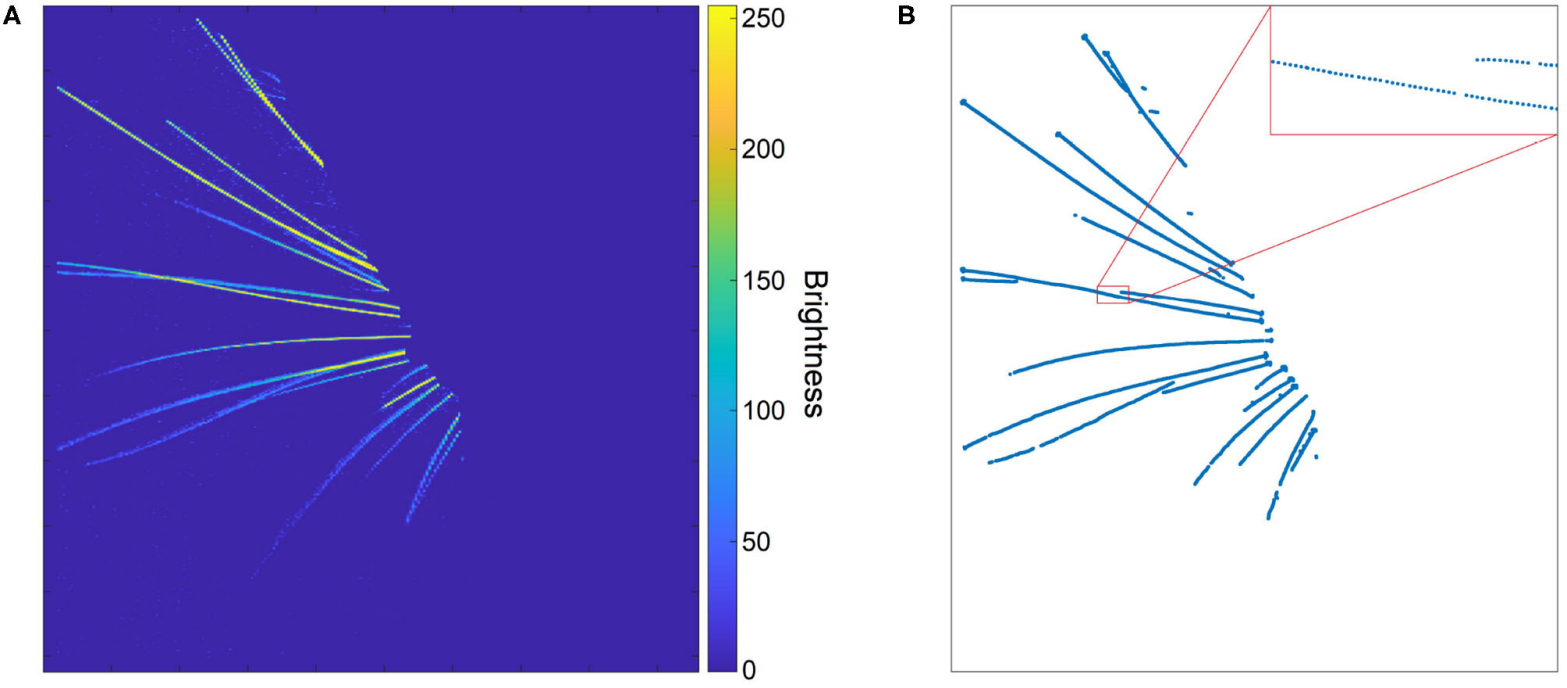
**(A)** A whisker image as height map after removal of background and silhouette. The whiskers appear as bright “ridges” in a dark “landscape.” **(B)** Whisker centerline points, obtained from the height map in **(A)** with the centerline detection algorithm. The inset shows that, even though the whiskers are not wider than a few pixels, the extracted centerline is rather smooth.

WhiskEras tracks whisker movement in three phases: pixel-level processing, parameter fitting and tracking, as shown in Figure 1. Each phase consists of multiple steps. The first phase, pixel-level processing, starts with background removal. This procedure was retained from BWTT and described in detail in Perkon et al. (2011). As the rest of WhiskEras, this procedure was written in MATLAB (MathWorks, Natick, MA, USA). As whiskers appear as dark objects on a bright background, the background was identified as the brightest pixels detected in several frames taken from across the video. The pixels were compared on a per-pixel basis, and the lightest ones were retained. This process filtered out everything except the whiskers and most of the fur, as in Figure 2B. Subtracting the background from the original frames made the whiskers appear bright on a dark background, as shown in Figure 3A. The mouse silhouette and undetected fur were removed by binarizing the image and performing a series of image-processing operations: dilation (implemented with the MATLAB function imdilate) and erosion (implemented with the MATLAB function imerode) as shown in Figure 2D.

The subsequent steps of BWTT, noise-filtering and detection, were not retained. Instead, WhiskEras considers whiskers as curvilinear structures as their width is negligible compared to their length. Therefore, extracting the position of the centerline of the whiskers is an abstraction that captures the essentials of whisker position and shape. Curvilinear structures appear in a wide range of images - not only whiskers, but also roads on air photos or endoplasmic reticular networks on electron microscopic images of cells. Steger's curvilinear-structure algorithm (Steger, 1998) solves the problem of detecting such structures and finding their centerlines in an elegant analytical way, by viewing the image as a height map, in which the color value represents the height. This principle is shown in Figure 3A. The whiskers can be considered “ridges” in a dark “landscape.” The cross section of such a ridge can be approximated by a parabola, which can be approached by a second-degree Taylor polynomial. The vertex of the parabola can then simply be found analytically by calculating the position where its derivative equals zero.

The algorithm, explained in detail in Steger (1998), can be performed on every pixel independently, which allows a very high degree of parallelism. Our own implementation of this algorithm combines MATLAB's gpuArray and arrayfun functions, allowing the algorithm to be performed on each pixel in parallel.

**Figure 4:**
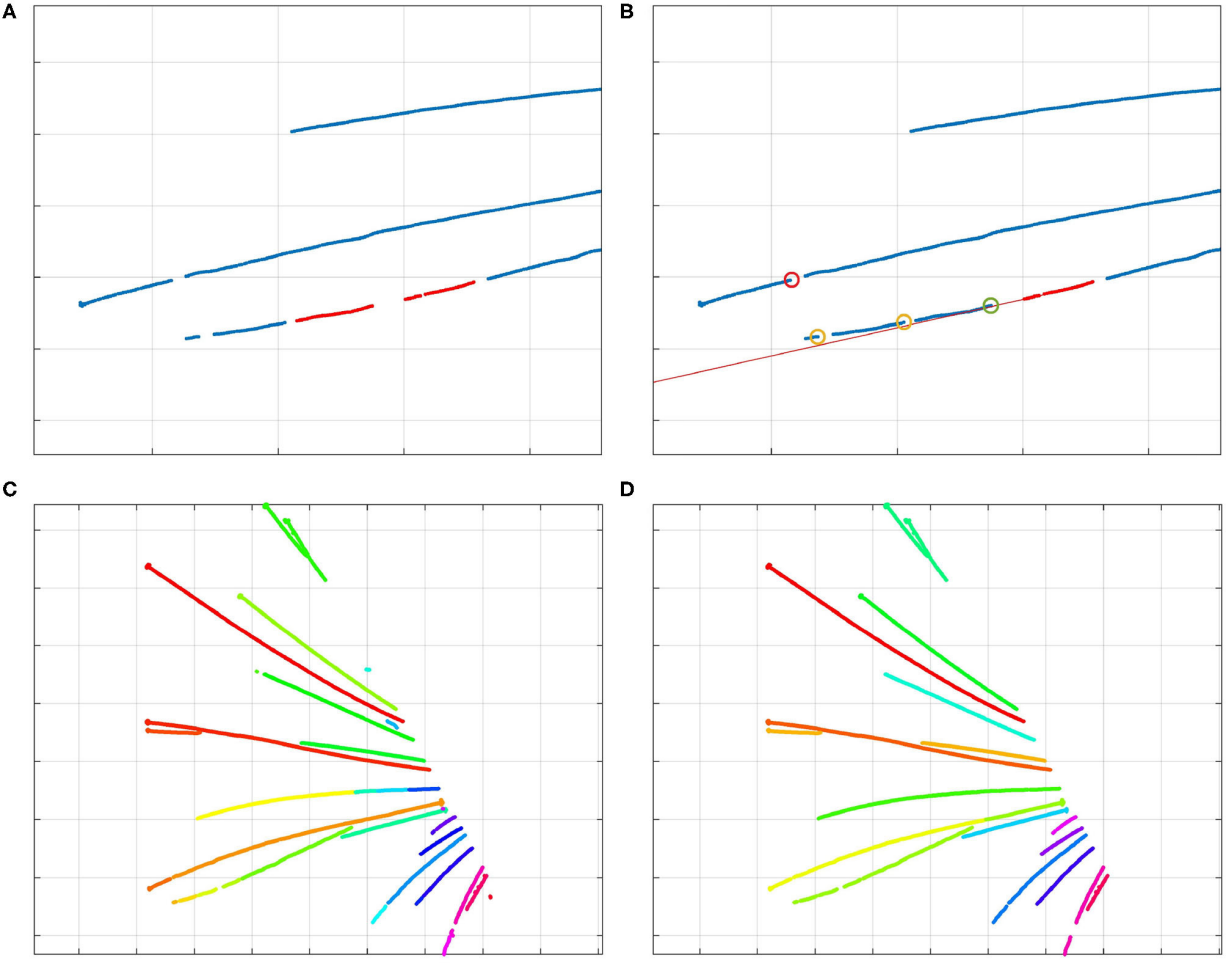
**(A)** The two red clusters of centerline points are part of the same whisker, and will be stitched by the algorithm. **(B)** Visualization of the stitching algorithm. The red line through the selected cluster (in red) shows the direction of the whisker; the bottom points of other clusters are marked by circles. The red circle signifies a bottom point that is too far from the line; therefore, that cluster should not be stitched to the selected cluster. The yellow circles signify bottom points of clusters that could be stitched to the selected cluster. However, the bottom point marked by a green circle is the best option. Therefore, the clusters are stitched together as was shown in **(A)**. By iterating over all the clusters, those that are part of that whisker will be stitched together by the algorithm. **(C)** Different colors signify different clusters of centerline points. Whisker clusters before applying the stitching steps, but after DBSCAN clustering. **(D)** Clusters after the stitching steps. Even the points of the long, crossing whiskers in the middle of the image have been clustered correctly, as the clusters were collinear.

The centerline points form dotted line segments rather than collections of pixels (Figure 3B), which makes it relatively straightforward to cluster them into actual whisker segments. It also allows for distinguishing crossing whiskers, as the upper whisker often has a different intensity than the lower whisker. In that case, the curvilinear structure detection algorithm manages to detect the centerline of the upper whisker in an uninterrupted fashion, whereas the lower whisker will be detected as an interrupted line segment. After all, the upper whisker will have a continuous centerline, whereas the centerline of the lower whisker is interrupted (Figure 4). Furthermore, the algorithm consists of a convolution step to find five partial derivatives of the frame, and a pixel-based calculation step in which the position of the line is determined. Both operations can be executed for each pixel in parallel, which reduces the execution time per frame. Figure 3B shows an image in which the centerline points of a set of whiskers are detected. Even though whiskers are no thicker than a few pixels, the centerline points form a rather smooth curve, as a result of the sub-pixel precision. Since the color and thickness of whiskers do not vary considerably, these centerline points of the whiskers, with their direction and intrapixel position, will serve as input to the parameterization step, whereas the rest of the information can be discarded.

### 2.3. Parameter Fitting

The parameter fitting process consists of two consecutive steps. First, centerline points are clustered so that each cluster corresponds to one whisker. Next, for each cluster, a curve is constructed that describes the shape and position of this whisker with a few parameters. These parameters should satisfy the compactness criterion: whiskers that are very similar should have similar parameter values, whereas whiskers that are very different should be very different in their parameter values (Duin and Tax, 2005). This two-step approach was chosen, as fitting curves directly to a large set of points is too computationally intensive: it is an NP-hard problem (non-deterministic polynomial-time) which is complicated even further by the fact that the number of whiskers on the frame is not known beforehand. After clustering, the problem of finding the best fitting curve per whisker is a regression problem, which can be solved by least-squares fitting. Moreover, after clustering, the whiskers can be processed in parallel, which reduces the processing time.

#### 2.3.1. Clustering

WhiskEras contains two different algorithms for clustering. The method of choice depends on the properties of the video recording. WhiskEras offers the user the option to choose one of the two methods. The first algorithm is DBSCAN (Ester et al., 1996). With this algorithm, the decision whether two points are part of the same cluster is taken based on the density of points in the region between the two points: if the two points are separated by a region with a high density, the points are likely to be part of the same cluster, whereas a region with sparse density suggests that the points belong to different clusters. This makes density-based clustering very suitable for the detection of long or irregularly shaped clusters, such as whiskers. If the distance between two points is smaller than a preconfigured parameter, they are part of the same cluster. A disadvantage of this approach is that it does not take into account that each centerline point has, at most, two neighbors. It is possible to build into DBSCAN a restriction on clustering to the two closest neighbors only, but this fails when the nearest neighbors are both on the same side. This regularly happens as each pixel contains at most one centerline point, which can potentially show up at any sub-pixel location.

Alternatively, a local clustering algorithm that builds upon the detection algorithm can be used (Steger, 1998). The algorithm makes use of the fact that the same information that is used to detect the sub-pixel position of the whisker point, can be used to detect the direction of the line at that particular point. If the local direction of the whisker is known, it is possible to search in that direction to find the next centerline point. In Steger's original clustering algorithm, each centerline point searches for one neighbor in its adjacent pixels, depending on the local direction. We expanded this, having each point to search for two neighbors in opposite directions, not only in immediately adjacent pixels, but also one pixel further. This allows for correct clustering, even if there is a gap because of a missing point. Centerline points are only clustered if they mutually classify each other as neighbors. This eliminates the problem of the clustering of more than two neighbors in DBSCAN. However, this system is heavily dependent on the accuracy of the determined local direction of the line - an error in either of the two neighbor points can inhibit proper clustering. The accuracy is highly dependent on the quality of the recording, and in our experience, Steger's clustering algorithm works best in cases where the detected centerline is smooth and uninterrupted, whereas DBSCAN works better in cases where the centerline is interrupted or not smooth (which occurs in lower-resolution recordings). Therefore, we decided to include both algorithms in the system, so that the user can decide on a per-case basis.

As illustrated in Figure 4C, after the first clustering step, the number of clusters can still be larger than the number of whiskers. This can happen because of whisker occlusions, or the failure to recognize whisker centerline points during the detection step. Therefore, it is necessary to reduce the number of clusters by merging those that are part of the same whisker together, in such a way that every cluster represents a single whisker.

Since initial clusters were already created in the previous step, we can determine their direction. After all, clusters that contain a certain number of points form line segments, and clusters that are part of the same whisker are usually more of less collinear. This collinearity can be used to decide which clusters belong to the same whisker. Therefore, we designed a cluster-stitching algorithm, which can be described as follows: a section of a certain length from the tip of a cluster *a* is approximated by a line *l*. This line is extended upwards, and all the clusters of which the bottom points come close to this line are marked. The cluster *b* of which the bottom point comes closest to the tip of the cluster *a*, can be merged with cluster *a*. This procedure can be repeated for every cluster long enough to be linearized. In this way, clusters that are part of the same whisker are stitched together. The procedure is illustrated in Figure 4. As can be derived from the colors in Figure 4D, every cluster now corresponds to one whisker. The algorithm also created a correct clustering of the crossing long whiskers.

A separate problem is the case in which whiskers fully overlap at their bottom, but diverge at their tips. In such cases, the centerline of the lower whisker appears to be disconnected from the snout after detection. We resolved this by copying the detected centerline points of the upper (visible) whisker and adding them to the cluster of the lower whisker, thereby making sure that both whiskers are connected to the snout and can be parameterized properly.

#### 2.3.2. Parameter Fitting

**Figure 5:**
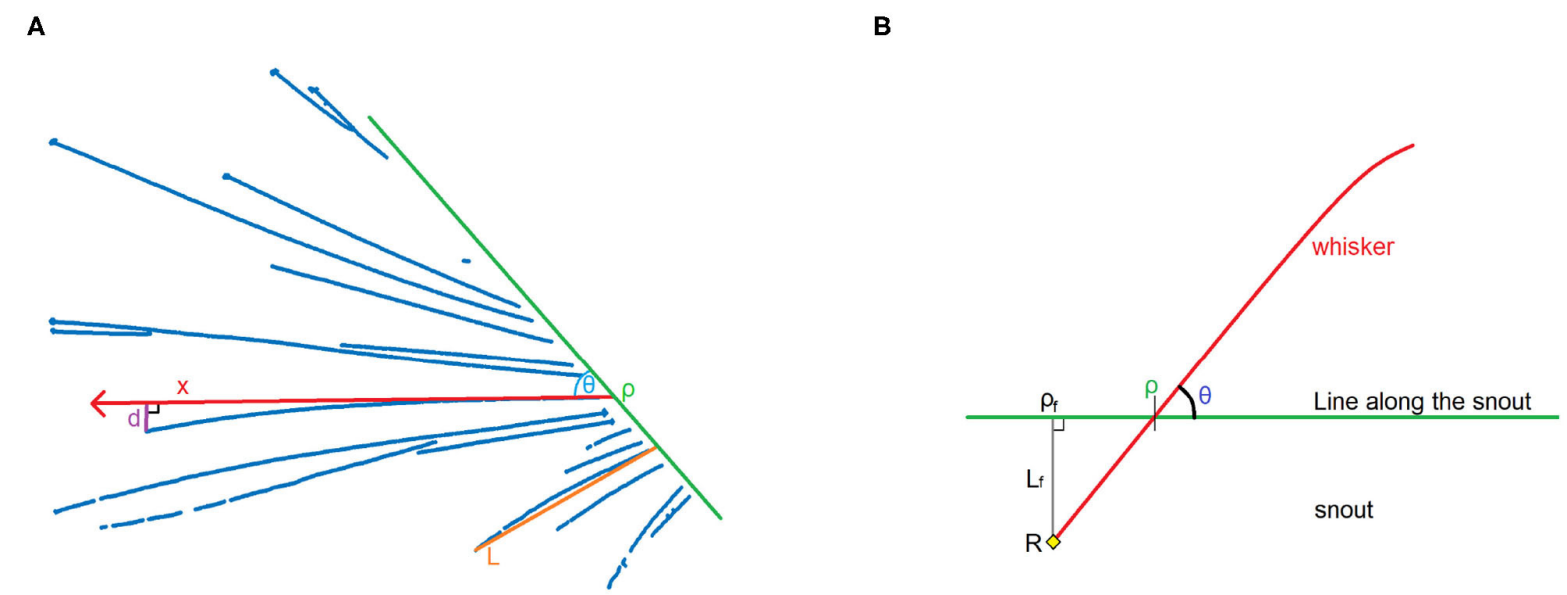
**(A)** Construction of the four parameters that are used to define a whisker. *d* is the distance between a line with its origin in ρ and angle θ, and the actual whisker, at point *x* on the line. Assuming that a second degree polynomial can be a good approximation of the actual shape of the whisker, *d* can be defined as *d* = *bx*^2^. Length *L* is determined as the difference between the tip and the bottom of the whisker (here shown on a different whisker). **(B)** Schematic image showing the relationship between position ρ and angle θ. When we assume that the position of pivot point *R*, and hence ρ_*f*_ and *L*_*f*_, are fixed, we can prove that ρ and θ are dependent variables. This can be used to recognize the same whisker in different frames.

A good way to perform the second step is by using parameters for characteristics that are similar for similar whiskers, and dissimilar for dissimilar whiskers. These characteristics are the angle to the snout, position, shape, and length. The angle can be quantified by drawing a line along the snout and measuring the angle θ relative to the line and position ρ on the line, as shown in Figure 5. The shape of the line can be approximated by a second degree polynomial, and therefore by one parameter *b*, where the distance *d* between a straight line and the actual whisker at a distance *x* from the snout is defined as *d* = *bx*^2^, as shown in Figure 5A. The length of the whisker can be approximated by measuring the distance between the bottom and the tip of the whisker, and defining this as length *L*. In this way, it can be said that the whisker is a function of the parameter tuple {ρ, θ, *b, L*}. These parameters can be fit to each cluster by using the MATLAB function nlinfit, in order to obtain an accurate representation of the whisker.

### 2.4. Tracking and Recognition

WhiskEras combines tracking and recognition, and is loosely based on the “Tracking-Learning-Detection” (TLD) algorithm described in Kalal et al. (2012). The main idea behind our algorithm is that, most of the time, tracking whiskers is relatively straightforward: the positions and shapes of whiskers usually only change slightly between frames; if the parameterization was done correctly, whiskers on frame *n* + 1 can be matched to those on frame *n*. Nevertheless, when whiskers move fast, cross, and hide, it is certain that the tracker, at some point, will make a mistake. In mice with an intact set of whiskers, it is almost impossible to recover from such a mistake. In such cases, it is useful to have a recognizer that can recognize a particular whisker based on its appearance, rather than its current position. In WhiskEras, the recognizer uses the data from the tracker to learn to recognize a whisker. In this way, WhiskEras can recover from tracking mistakes.

**Figure 6:**
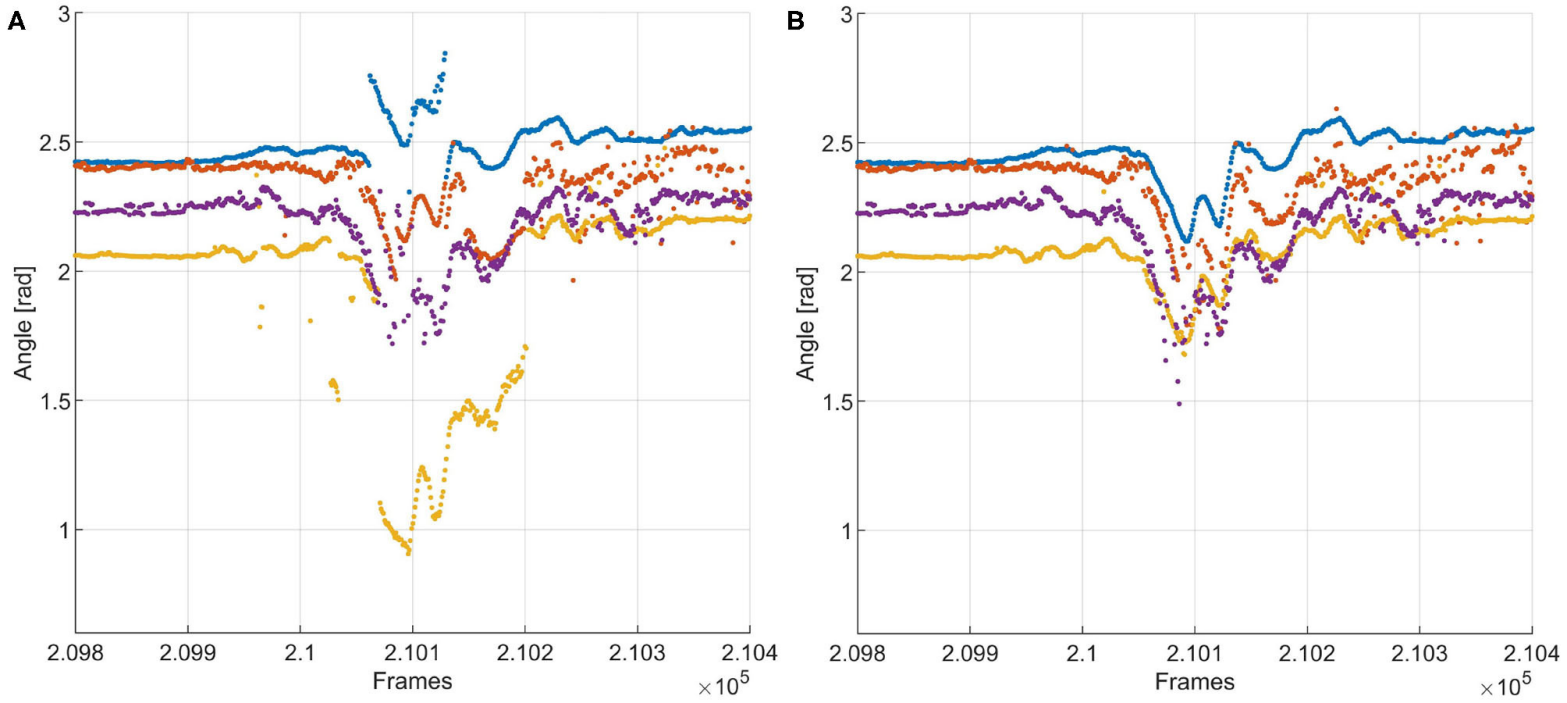
**(A)** Tracking of a whisker fragment with N- and P-experts turned off. The red and yellow tracks contain jumps of more than 10 degrees in whisker angle between two frames—i.e., within 1 ms, since the video was recorded with 1,000 Hz. This is physically impossible, so this is the result of incorrect recognition. **(B)** Tracking results of the same fragment, with the experts turned on. Incorrect recognition of the blue and yellow traces is detected by the N-expert, after which the P-expert assigns whiskers to those tracks that are most likely correct. The corrected tracks are added to the training data for the recognizer, aiding it in preventing such mistakes in the rest of the fragment.

Accumulation of tracking and detection errors in the training data could lead to progressive deterioration of the training data and therefore of the recognition mechanism. This is countered by introducing a set of two auxiliary algorithms aimed at checking the work of the recognizer and tracker by “experts” (Kalal et al., 2012): an “N-expert” that checks whether a recognition or tracking decision is feasible, given constraints in the changes in whisker position between two consecutive frames. In addition, a “P-expert” considers whiskers that were parameterized but not matched with any whisker in the previous frames, and tries to match the “orphan whiskers” with whiskers from the previous frames that could not be matched with any of those in the current frame. Even though the experts themselves are not immune to mistakes, if the number of mistakes in the training data remains limited, progressive deterioration becomes less likely. The effect of expert correction is illustrated in Figure 6.

In WhiskEras, we implemented a version of TLD that starts with a “bootstrapping” period with a variable length that can be defined by the user. In this period, the classification is done by a tracker that predicts the characteristics of a whisker on the next frame by means of a Kalman filter, and matches predictions of the tracked whiskers with detected whiskers on the new frame. This will create an initial training set for the recognizer and works best if the whiskers do not move too fast during the bootstrapping period.

Recognition of whiskers in WhiskEras is essentially a classification problem: we want to distribute all detected whiskers in all frames into groups, each representing the same whisker. As a recognizer, we chose a Support Vector Machine (SVM), “one-vs-one” approach. As parameters, we use the length *L*, bending parameter *b*, position ρ and the cotangent to of the angle θ. We also use the difference between the mean of ρ and cotθ of the previous frame, and the value of θ and ρ of the current whisker as values (we use the mean value from the previous frame, as the mean value for the current frame can only be reliably calculated when all the whiskers are identified). We use cotθ instead of θ as this helps us to approximate the pivot point of the whisker (as will be explained below). Although this point is not fixed, it is relatively stable between consecutive frames and can therefore be used as recognition point for whiskers over consecutive frames.

On the videos, the pivot point of the whisker is not visible, as it is hidden by the silhouette of the mouse. Nevertheless, if the line along the snout, which is used to determine ρ and θ, is chosen well, and the whisker changes its angle over multiple frames, it is possible to determine the pivot point with good accuracy, in the following way:

Let ρ be the position on the line along the snout, and θ the angle of the whisker relative to this line (Figure 5B). Everything proximal to the line along the snout will not be detected, but it is known that the whiskers we see are connected to the snout. It will be assumed that the part of the whisker close to the snout is more or less linear (which is usually the case; whiskers are more bent at the top since they are tapered, Williams and Kramer, 2010). We can construct a line perpendicular to the snout that crosses the pivot point *R*. Let ρ_*f*_ be the point where this line intersects with the line along the snout, and *L*_*f*_ be the distance between *R* and ρ_*f*_. This will create a right triangle ρ_*f*_ρ*R*. Because we assume a linear whisker situation near and below the line along the snout, the angle ∠ρ_*f*_ρ*R* = θ.

According to trigonometry, $\cot\theta =\frac{\rho -\rho_{f}}{L_{f}}$. This leads to the equation:

(1) $$\begin{array}{l} \rho =L_{f}\cot\theta +\rho_{f} \end{array}$$

We assumed *R* to be a fixed point, therefore *L*_*f*_ and ρ_*f*_ are constants. Equation 1 therefore represents a straight line *Q* of the form *y* = *ax* + *b*. If we detect ρ and θ for the same whisker in different positions, it is possible to estimate *Q* by plotting cotθ against ρ. With a sufficient amount of training data, an SVM can use the value of cotθ relative to ρ_*f*_ for classification.

The tracker follows the whiskers that were detected on the first frame. After the bootstrapping period, the classifier is used first to recognize whiskers in a frame. The N-expert determines the difference in θ and ρ between frame *n* and *n* − 1 for each whisker, and determines whether it is plausible that a whisker changed its position by that amount in this short time interval. If a whisker position in frame *n* is too dissimilar from its counterpart in frame *n* − 1 (i.e., its position (ρ, θ) changed more than is possible between two frames, based on a threshold empirically determined and predefined by the user), the N-expert marks the detection in *n* as a false positive and removes it from the training data and the output.

The tracker now takes the role of P-expert: it tracks whiskers from frame *n* − 1 to frame *n*. If it assigns an identity that was not used by the classifier to a whisker that was not classified by the classifier, this is marked as a false negative. The whisker is added to the training data and the output. If a whisker remains undetected by both the classifier and the P-expert, its position is estimated. The P-expert can use this estimation to redetect the whisker in later frames, but the estimation will not be part of the output.

The classifier consists of $\frac{\left(N\right)\left(N-1\right)}{2}$ SVMs (with *N* the number of whiskers that is being tracked, i.e., the whiskers that were visible on the first frame) that are retrained in parallel every *s* frames, with *s* being another parameter that can be configured by the user. The training data consists of all classified whiskers from frames *n* − *W* to *n*, with *W* being the window size, which can also be configured by the user. The MATLAB function fitcsvm is used to train the SVMs. The parameters sets are standardized to a mean of 0 and a standard deviation of 1.

### 2.5. Comparison With Other Trackers

We compared our tracker with two widely used trackers: BWTT and Janelia Whisk, by processing four video fragments, each 100,000 frames in length. We used Ma's accelerated version of BWTT (Ma et al., 2017), and version v1.1.0d of Janelia Whisk, downloaded from their Wiki (Clack, 2011a).

Even though all three tools are aimed at tracking whiskers, their purposes are slightly different. BWTT has been designed to produce the average angle and position of the whiskers. For each of the frames, it tries to detect as many whiskers as possible, and calculates their average angle. Individual whiskers are not tracked over time. False positives are avoided by only detecting whisker shafts in a narrow band around the snout in which there are only whiskers, but no fur hairs or other objects. Janelia Whisk has been designed to detect and track manually selected whiskers. There is not much protection against false positives, and in videos, artifacts were often falsely detected as whiskers if the “trace all curves” feature is used. Because it is possible to manually deselect false positives, this is not much of a problem for the user. However, we have to take the differences between the different tools into account when comparing their quality.

We designed four metrics to compare the different trackers. The first metric is the number of detected whiskers per frame. Here, we will only compare WhiskEras to BWTT, as Janelia Whisk detects (by design) more false positives than the other two trackers, which makes this metric not usable for that tracker. The second metric is the detection ratio per whisker, i.e., the number of frames in which the whisker was recognized as a percentage of the total number of frames. Here, only WhiskEras and Janelia Whisk will be compared, as BWTT is not capable of tracking individual whiskers in untrimmed mice. The third metric is the Signal-to-Noise ratio of the detected whisker traces, which gives an indication of the precision of angle detection. The Signal-to-Noise ratio is the ratio of the power of a signal to the power of the noise. Again, we will only compare WhiskEras and Janelia Whisk, as BWTT does not track individual whiskers. Lastly, we will assess the tracking quality of the two trackers that are capable of tracking individual whiskers.

We first processed the fragment using BWTT. Then, we processed the fragments with WhiskEras. Finally, we processed the videos with Whisk. In Whisk's GUI, we selected the same whiskers for tracking as in WhiskEras (when possible), the criterion being their length and visibility on the first frame. The parameters of WhiskEras, such as the clustering method used, were determined and adjusted based on trials runs on the first few frames. For Whisk, we followed the steps listed in the Automated Whisker Tracking tutorial, which can be found online (Clack, 2011b). However, it does mean that the number of automatically detected whiskers per frame is not a good metric to assess the quality of the tracker. WhiskEras attempts to detect all the whiskers. It does not yet support manual whisker selection (although this can be implemented in a later version), and distinguishes fur and whiskers based on their length. Static artifacts are filtered out by the background removal algorithm.

## 3. Results

**Figure 7:**
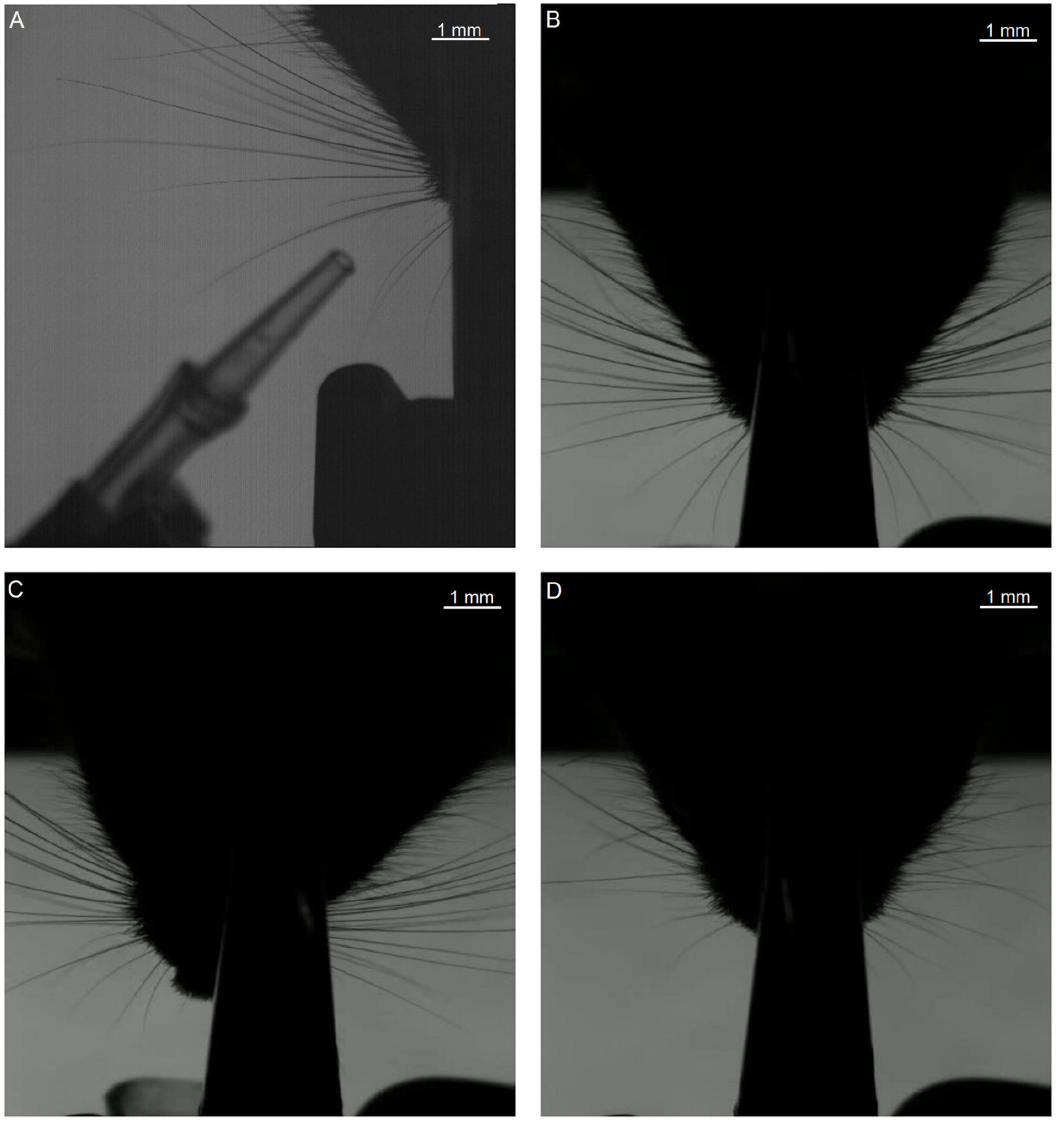
First frames of each of the segments that were used in testing. The letters **(A–D)** of the subfigures correspond to the video labels. Segment A shows only the right side of the mouse. The other segments show both sides, but we only tracked the subjective right side (i.e., the left side on the image).

Assessing the quality of the output of WhiskEras, or any other whisker tracker, is not a trivial task as there is no “perfect” output available for comparison. The fast movements, occlusions and crosses of whiskers make individual whiskers also difficult to recognize over time for a human observer. We therefore chose to compare the output of WhiskEras with those of two established whisker tracking. To this end, we used four video segments with a length of 100,000 frames each (Table 1, first frames of each video shown in Figure 7).

**Table 1:** Specifications of the videos that were used.

**Label**	**Recording date**	**Resolution (pixels)**	**Frequency**	**View of mouse**	**Camera**
		**(pixels)**	**(frames/s)**		
A	08-06-2017	480 × 512	1,000	Right side of snout	Basler A504k
B	09-09-2019	480 × 512	750	Full snout	Basler acA640-750um
C	09-09-2019	480 × 512	750	Full snout	Basler acA640-750um
D	10-09-2019	480 × 512	750	Full snout	Basler acA640-750um

### 3.1. Video Fragments

For this analysis, focused on the whiskers on the right of the snout. The fragments each have their own challenges when it comes to recording: fragment A features a prominent non-mystacial whisker; fragments B and C show a relatively large number of whiskers. Fragment D shows fewer whiskers, but features a non-mystacial whisker, which crosses the mystacial whiskers.

### 3.2. Detected Whiskers per Frame

A simple metric to describe the reliability of whisker tracking is the average number of tracked whiskers per frame. As mentioned in section 2.5, we only compare WhiskEras to BWTT for this metric.

BWTT simply attempts to detect all the whiskers in a narrow band around the snout. WhiskEras detects all whiskers, but gives the option to exclude some based on length. Furthermore, the silhouette removal algorithm can be adjusted to remove more of the fur—this reduces the chance of fur incorrectly being labeled as whiskers, but it increases the chance that shorter whiskers are missed. For this metric, we set the minimum length to a number that prevents fur from being misclassified as a whisker.

**Figure 8:**
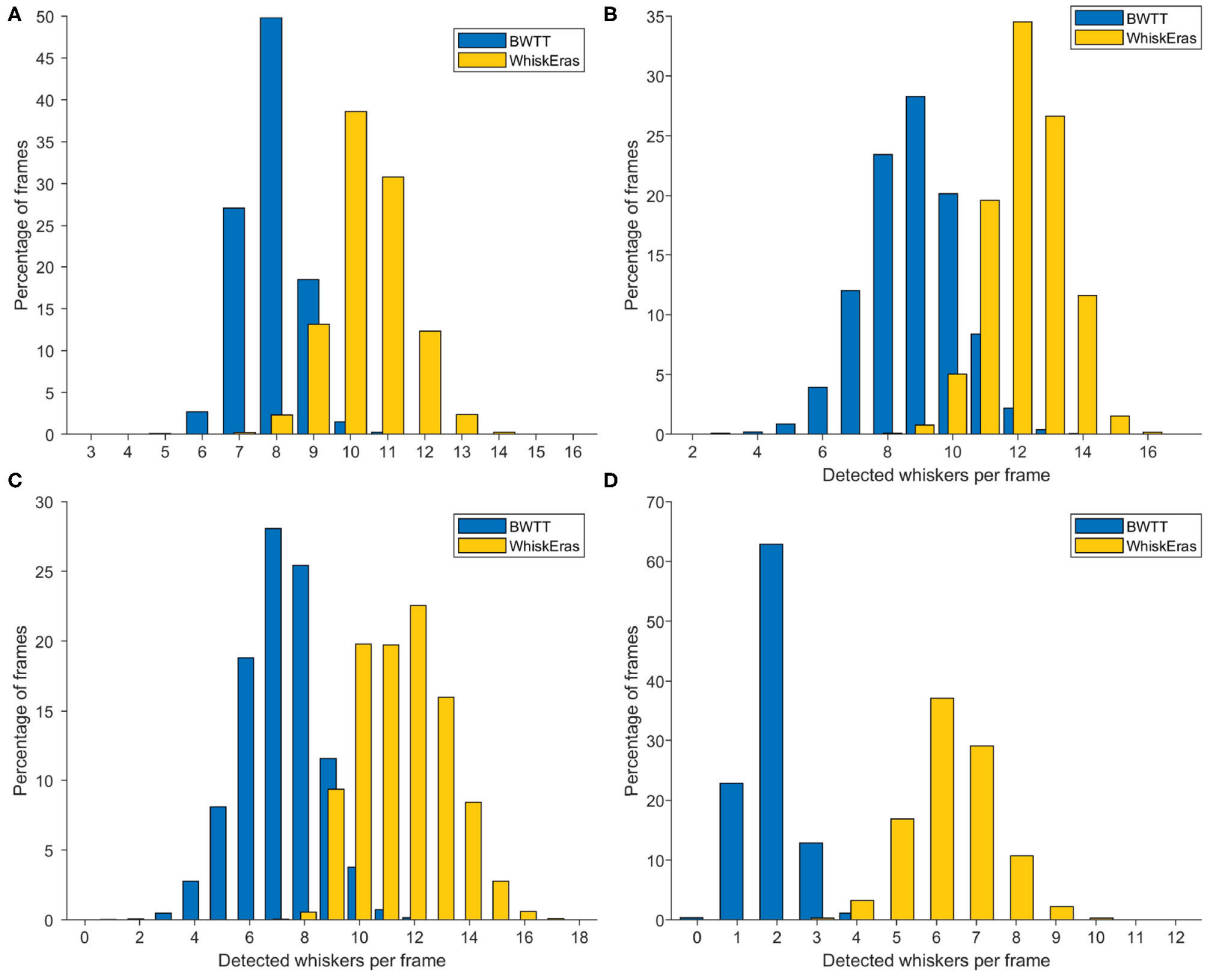
Histograms of detected whiskers per frame for each of the videos. The letters of the subfigures correspond to the video labels. In each video, WhiskEras detected on average more whiskers per frame (all *p* < 0.001, paired t tests). The averages and standard deviations of the number of detected whiskers per frame for each video are as follows: **(A)** BWTT: μ = 7.89, σ = 0.81, WhiskEras: μ = 10.45, σ = 1.05, **(B)** μ = 8.81, σ = 1.44 / μ = 12.22, σ = 1.15, **(C)** μ = 7.18, σ = 1.43 / μ = 11.54, σ = 1.61, **(D)** μ = 1.92, σ = 0.64 / μ = 6.34, σ = 1.10.

The results are shown in the histograms in Figure 8. It becomes immediately clear that on this metric, WhiskEras outperformed BWTT: it consistently detected more whiskers. Whereas BWTT only detected short whisker shafts at a specific distance from the snout, WhiskEras tried to find the whiskers everywhere on the desired side of the snout, thus detecting shorter whiskers and whiskers that are partially occluded. On average over all four videos, WhiskEras detected 90.7% more whiskers per frame than BWTT.

### 3.3. Detection Ratio

The detection ratio is determined for individual whiskers: it is the percentage of frames in which the particular whisker was detected. Even though this metric can give an indication as to how well the tracker was able to follow whiskers over time, it does have the risk of false positives: a tracker can label a particular whisker as “detected,” whereas in reality, it has detected a different whisker or an artifact.

For this metric, the tracker needs to be able to track individual whiskers over longer periods of time, so we cannot include BWTT into this comparison. We tracked the same whiskers in WhiskEras and Whisk. For Whisk, there was the limitation that the software had trouble processing our very long sequences of frames. Therefore, we cut up each of our videos in five segments of 20,000 frames, and processed them as if they were separate videos. Since the first frame of each segment is used to select whiskers for Whisk to track, and not all whiskers are visible all the time, we could not track all whiskers in all the segments with Whisk. In such cases, we only considered the segments in which the whiskers could be tracked by both Whisk and WhiskEras.

The results for every tracked whisker in every video are summarized in Table 2. It can be seen that Janelia Whisk, in general, scored higher on this metric than WhiskEras. This is especially the case for video A, for which Janelia Whisk reports detection ratios of more than 99% for every tracked whisker. For WhiskEras, the detection ratios in the best whiskers are similarly high, but it found some other whiskers more difficult to track. The only video where WhiskEras has a higher average detection ratio than Janelia Whisk, is video D.

**Table 2:** Detection rate per whisker for Janelia Whisk and WhiskEras.

**Whisker no**.	**Video A (%)**	**Video B (%)**	**Video C (%)**	**Video D (%)**
1	99.86/91.67	100.0/61.45	95.59/99.78	93.71/94.47
2	99.93/99.92	100.0/87.56*	97.11/99.15	98.83/92.53
3	99.93/99.64	100.0/75.23	97.20/99.26	98.65/94.22
4	99.93/81.45	100.0/78.98*	97.20/99.58	80.08/95.75
5	99.93/69.81	99.99/64.10*	97.20/98.24	
6	99.93/99.79	100.0/86.92	96.90/94.18	
7	99.76/98.54	99.99/85.92	97.18/92.42	
8	99.88/98.78	99.97/61.94	96.02/90.61	
9	99.29/99.62	99.89/96.67	84.33/78.44	
10		99.35/99.57		
11		98.29/82.87		
12		89.88/85.85		
13		76.86/97.57		
14		55.99/73.01		
Avg.	99.83/93.25	94.30/81.26	95.41/94.63	92.82/94.24

*The first number of each entry is the detection ratio of Janelia Whisk, the second one is the detection ratio of WhiskEras. The whiskers for which Janelia Whisk was unable to track the whisker in one or more segments are marked with an asterisk*.

The lower detection ratios for WhiskEras could be due to the fact that our tracker is rather strict when it comes to false positives: the “N-expert” assesses whether a particular match is feasible, and if it is not, the match is marked invalid. For the WhiskEras recognizer, it is important that the training data be kept as clean as possible. Janelia Whisk, on the other hand, tries to find every whisker in every frame, which leads to very high detection ratios for most whiskers. However, this also comes with a higher risk of false positives.

### 3.4. Signal-to-Noise Ratio

The signal-to-noise ratio (SNR) can be computed as the ratio of the summed squared magnitude of the signal and that of the noise. Since the frequencies of our videos (750 and 1,000 Hz) are much higher than the frequency at which whisking occurs (up to around 30 Hz), we can visualize the actual movement of the whiskers by smoothing the measured signal. The variations in angle that occur at a higher frequency can be considered noise which originates from the tracker. To smooth the signal, we used MATLAB's smoothdata function. For smoothing, we chose the Savitzky-Golay filter, which smooths using a quadratic polynomial which is fitted over each window (MATLAB, 2020). We chose a window size of 10 frames. The noise is then approximated by subtracting the smoothed signal from the original measurements, after which we used MATLAB's snr function. The higher the SNR value, the less noisy the signal is. Since BWTT does not track individual whiskers, we only compared Whisk and WhiskEras.

**Figure 9:**
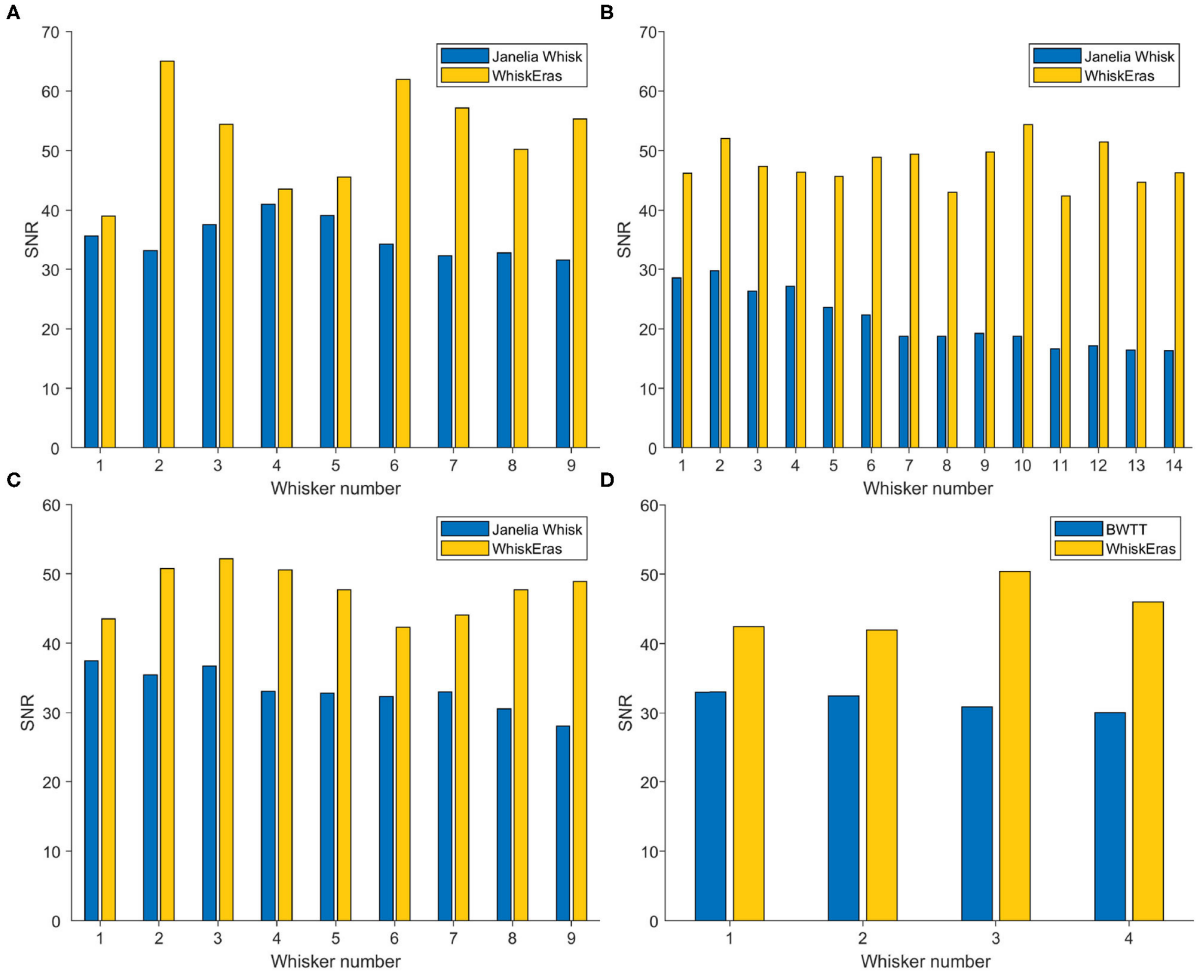
Bar diagram of SNR (in dB) of each whisker for each video, as tracked by WhiskEras and Janelia Whisk. The letters of the subfigures correspond to the video labels. A higher SNR signifies a higher precision of angle detection. WhiskEras consistently reports a higher SNR, with the following average SNR values over all whiskes: **(A)** Janelia Whisk 35.26, WhiskEras: 52.46 **(B)** 21.40/47.70 **(C)** 33.30/47.54 **(D)** 31.60/45.15. The ranges of the SNR values for Janelia Whisk and WhiskEras are as follows: **(A)** [31.54 − 41.00]/[39.03 − 65.02] **(B)** [16.32 − 29.73]/[42.37 − 54.40] **(C)** [28.00 − 37.52]/[42.33 − 52.17] **(D)** [30.01 − 33.02]/[41.93 − 50.33]. We performed a paired *t*-test on each segment, with the following *p*-values: **(A)** *p* = 0.002, **(B)** *p* < 0.001, **(C)** *p* < 0.001, **(D)** *p* = 0.012.

The SNR values are determined for each whisker in each video, and are shown in Figure 9. Here, WhiskEras outperformed Janelia Whisk for every whisker in every video. In concrete terms, this means that WhiskEras's traces of the angle of single whiskers were more stable than those produced by Whisk. This makes it easier to track whiskers over time, as their trace is more predictable and less noisy. On average over all the videos, the SNR of WhiskEras was 64.3% higher than the SNR of Janelia Whisk.

### 3.5. Tracking Quality

Tracking quality can be measured by how well the tracker is able to consistently label the newly detected whiskers correctly. If the tracking and labeling are done well, individual whiskers can be distinguished without much effort from the user. Secondly, even when the tracker loses a whisker (which is sometimes inevitable, given that whiskers can be hidden on some frames), the tracker should be able to recognize the whisker and label it correctly, as soon as it reappears. We will use visual inspection here, to compare WhiskEras to Janelia Whisk, as we have no gold-standard “correct” traces. Again, because BWTT does not track individual whiskers, we will not consider that tracker for this metric.

**Figure 10:**
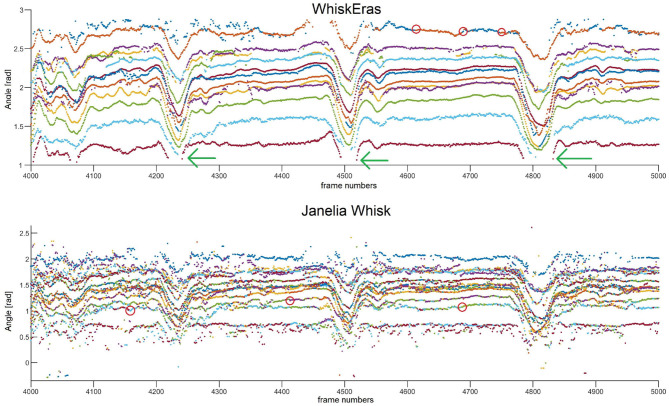
Whisker traces for video B, frames 4,000–5,000, as tracked by WhiskEras and Janelia Whisk. The y-axis shows the whisker angle in radians. Different colors denote differently labeled whiskers. It is clear that the WhiskEras trace is much clearer, and whiskers are more easy to follow and distinguish over time. There are some instances where the whisker traces suddenly switch color (we marked some of these with red circles). There, it is likely that the tracker made a mistake, as one would expect flowing lines. The green arrows show instances where a temporarily occluded whisker is found back by the recognizer when it reappears.

A trace of whisking behavior from video B is shown in Figure 10. This video is arguably the most challenging of the four, as it features the highest number of tracked whiskers. The segment consists of 1,000 frames, which corresponds to 1.33 s, in which the mouse whisks several times. More traces can be found in the Supplementary Materials. Overall, we found a strong congruence in the movement of all whiskers in line with the musculature of the whisker pad (Dörfl, 1985; Simony et al., 2010; Bosman et al., 2011; Haidarliu et al., 2015) and as also found in previous studies (Wolfe et al., 2008; Azarfar et al., 2018). The results are in line with our observations regarding detection ratio and SNR. The traces produced by Janelia Whisk appear very noisy, as the whisker angles vary a lot between individual frames. In contrast, most of the traces in WhiskEras appear very stable and easy to follow by eye. All the whiskers benefit from WhiskEras improved tracking quality. The higher performance of WhiskEras can be partly attributed to the higher SNR in the WhiskEras traces (which make the individual traces less noisy), but also to the fact that WhiskEras is able to find back whiskers after they have been temporarily occluded (as shown in Figure 10). This is very useful in videos of untrimmed mice, since occlusions happens regularly there.

With WhiskEras, the upper two traces (with the largest angle to the snout) are a bit more noisy than the other traces and appear to switch color from time to time, which is a sign that the tracker has difficulties distinguishing between the two. The lower trace (with the smallest angle to the snout) disappears each time the mouse whisks, but is redetected every single time. With Janelia Whisk, it is very difficult to distinguish between individual whiskers, as traces appear to switch colors all the time.

If we look at the overall picture of a longer trace of 20,000 frames (as shown in the Supplementary Figure 2), it becomes clear that WhiskEras can follow whiskers properly for thousands of frames. However, the longer the segment, the higher the chance that WhiskEras will confuse some of the whiskers without correcting; with video B, this happens after about 50,000 frames. Even though most of the other whiskers are unaffected, this leads to a decline in tracking quality as the training data becomes contaminated with errors. This is one of the explanations for the low detection ratio for some of the whiskers: when errors accumulate in the training data, recognition becomes more faulty. One solution to this could be to add an extra post-processing step to the algorithm for detecting such mistakes and corrected them, both in the actual trace and in the training data. Since these errors are easy to spot by eye in the trace, such an additional algorithm step is surely attainable, for instance by a postprocessing algorithm that checks for sudden jumps in whisker angle. As for tracking quality, WhiskEras had the best performance on the tested videos, and can be run on sizable video segments.

## 4. Discussion

We have developed a novel approach to track the movement of mouse whiskers without the need to trim or mark them first. In comparison to other, established whisker tracking algorithms, WhiskEras produces results with relatively low noise even during periods with rapid whisking.

Our algorithm consists of three main processing steps, which, in turn, consist of several smaller steps. This procedure allows us to effectively move from a raster image to an analytical representation of the whiskers. Such a pipeline is, however, vulnerable for errors in early steps that carry over in later steps. WhiskEras counters this potential weakness by the combination of tracking and recognition. While tracking aims to follow individual whiskers over time, it is vulnerable to the loss of a whisker in any given frame. Recognition, which is insensitive to time, enables recovery after such a loss. Using the combination of tracking and recognition, WhiskEras is able to reach a high signal-to-noise ratio and can track individual whiskers over tens of thousands of frames, even during periods of rapid movement.

With the rapid progress in artificial neural networks and deep learning, machine learning approaches to whisker tracking are likely to evolve in the near future. Currently, DeepLabCut shows promising results on behavioral tracking (Mathis et al., 2018; Mathis and Mathis, 2020). However, mouse whisker tracking remains a challenging task, whatever the approach, and people currently rely on whisker clipping when using DeepLabCut for whisker tracking (Dooley et al., 2020). We are convinced that both techniques, computer vision as used by WhiskEras as well as deep learning, are powerful tools and it will be interesting to see what the future brings; potentially even a merger of both techniques where computer vision contributes to the generation of labeled data required for DeepLabCut or similar programs.

A challenge for any movement tracker is to be flexible and be able to reliably track whiskers filmed under different conditions. We show here that WhiskEras can handle videos that differed in frame rate, pixel count and zoom. This flexibility is partly due to the large number of parameters that can be adjusted to optimize tracking. Based on empirical experience, standard values for each of these have been obtained.

Currently, WhiskEras is optimized for recordings of head-fixed, untrimmed, free-whisking mice. The whiskers do not touch objects, which allowed us to describe the shape of a whisker by one single parameter, and the length by another. When objects are included, the whisker will be deformed, its shape depending on the place of the object and the length of the whisker. To allow tracking in those circumstances, the set of abstraction parameters needs to be expanded. Furthermore, the background-removal algorithm relies on the background being static over the course of the video; if other moving objects are added to the background, these would need to be filtered out using an additional preprocessing step.

Whisker movements are not restricted to a single plane and several studies have focused on their 3D behavior (Bermejo et al., 2002; Knutsen et al., 2008; Petersen et al., 2020). As the mechanoreceptors at the base of the whiskers are organized in a 3D fashion (Rice et al., 1986; Bosman et al., 2011), a proper description of whisker movements in three dimensions leads to a more accurate understanding of whisker use. Potentially, adding a side view is also helpful to recognize individual whiskers. However, adding the third dimension to whisker tracking poses new problems and, currently, whisker tracking in 3D depends either on labels attached to whiskers (Bermejo et al., 2002) or on clipping of most whiskers (Knutsen et al., 2008; Petersen et al., 2020). As most of the movement takes place in one plane (Bermejo et al., 2002; Knutsen et al., 2008; Petersen et al., 2020), we chose to restrict WhiskEras to a 2D tracker at least for the time being.

Tracking speed of WhiskEras is not optimal yet, but we have previously shown with BWTT that an acceleration of a few thousand times is realistic (Ma et al., 2017). Also, the multitude of parameter settings that can be defined by the user makes the software complex to use at this point. To address both issues, work is already underway with porting of WhiskEras to lower-level programming languages, which is guaranteed to lead to high speedups.

**Figure 11:**
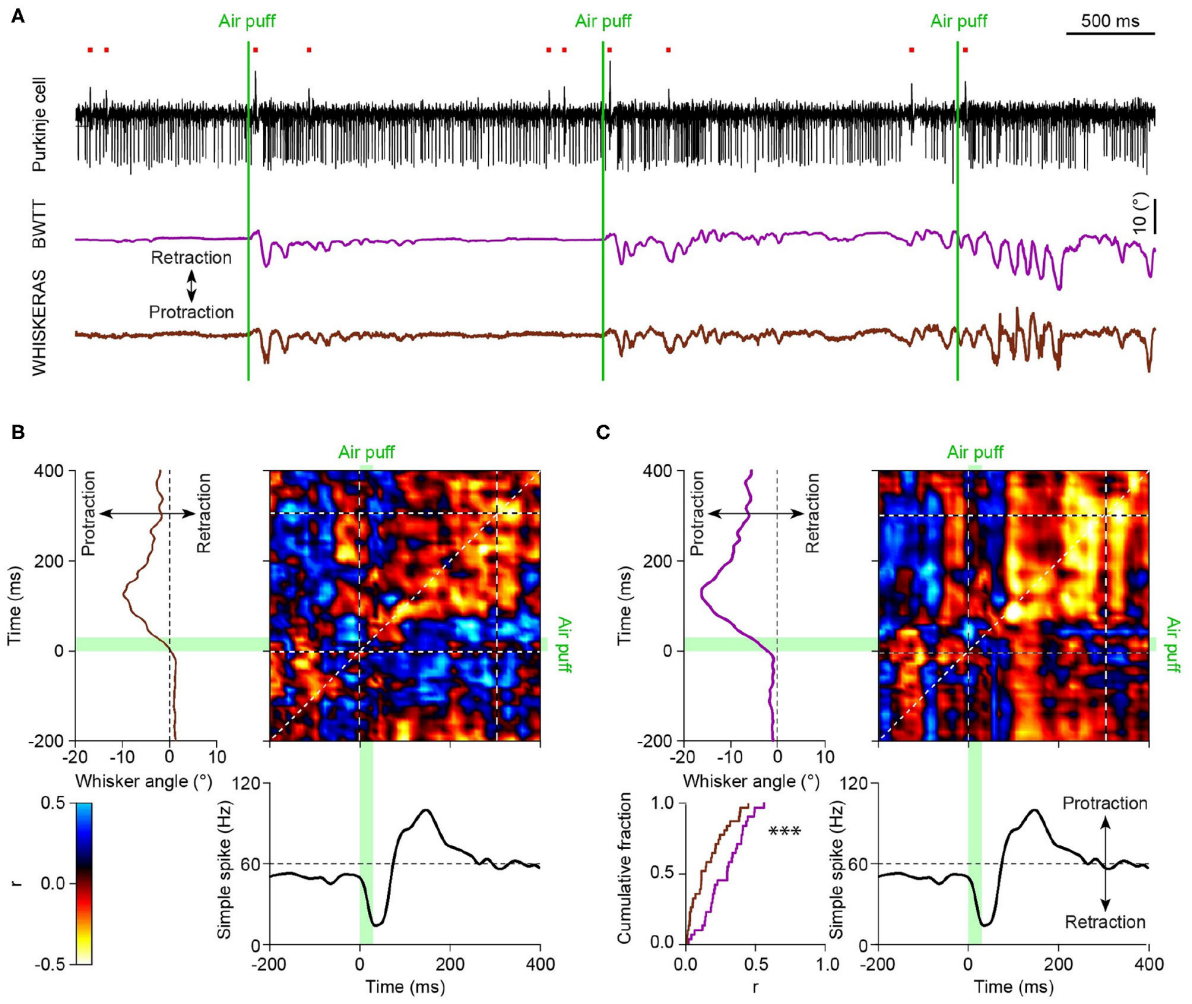
**(A)** Top: Exemplary trace of Purkinje cell activity recorded in an awake mouse with an extracellular electrode revealing complex spikes (upward deflections indicated by the red dots) and simple spike (downward deflections). Below are the unfiltered average whisker angles as obtained using BWTT and WhiskEras, respectively. **(B)** Matrix of correlation between the fluctuations in simple spike activity and the average whisker angle (processed using BWTT). On the top left the average whisker angle and, on the bottom, the instantaneous simple spikes frequency around the moment of the air puff stimulation. The heat map represents the Pearson correlation value (see scale bar). The green bars indicate the time of the air puff stimulation. **(C)** The same analysis based on the whisker angles as obtained using the WhiskEras shows a higher level of correlation between whisker position and simple spike activity (*p* < 0.0001, paired t test between the Pearson correlation coefficients on the diagonal of the matrices, using the 0–300 ms interval).

With each tracking method having its pros and its cons, in the end it is crucial to see whether WhiskEras provides more useful data than previous methods. To this end, we compared the output of WhiskEras to that of BWTT during an experiment during which we simultaneously recorded the activity of a cerebellar Purkinje cell. From this comparison, it is clear that the WhiskEras data shows a higher level of correlation between whisker position and spike activity (Figure 11).

## 5. Conclusion

The algorithm was implemented in MATLAB and tested on four video segments, each 100,000 frames long. We compared WhiskEras to two current state-of-the-art whisker-tracking applications, Janelia Whisk and BWTT. WhiskEras detects more whiskers than BWTT: for our videos, on average 90.7% more. WhiskEras tracks whiskers more accurately than Janelia Whisk: for our videos, the SNR of WhiskEras is 64.3% higher than the SNR of Janelia Whisk. For three of our four videos, Janelia Whisk shows a higher average detection ratio for individual whiskers than WhiskEras (averaged over all videos: 95.59% for Janelia Whisk vs. 90.85% for WhiskEras), but the tracking-quality metric showed that Whisk tends to show false positives (as can be seen in Figure 10 and the Supplementary Material), whereas WhiskEras rather reports a whisker as “undetected” when it cannot find it, in order to prevent the training data from becoming corrupted.

## Data Availability Statement

The raw data supporting the conclusions of this article will be made available by the authors, without undue reservation.

## Ethics Statement

All experimental procedures were approved a priori by an independent animal ethical committee (DEC-Consult, Soest, The Netherlands) as required by Dutch law and conform the relevant institutional regulations of the Erasmus MC and Dutch legislation on animal experimentation. Permission was obtained under license numbers EMC3001 and AVD101002015273.

## Author Contributions

This study was conceived by JB, ZA-A, CS, and LB. VR performed the mouse experiments and analyzed the electrophysiological data. WhiskEras was designed and tested by JB with contributions from VR, ZA-A, CS, and LB. CS and CDZ provided the funding. JB and LB wrote the manuscript with contributions from all authors.

## Conflict of Interest

The authors declare that the research was conducted in the absence of any commercial or financial relationships that could be construed as a potential conflict of interest.
