# Supplementary material for: WhiskEras: A New Algorithm for Accurate Whisker Tracking
Source: Front Cell Neurosci. 2020 Nov 17;14:588445. doi: 10.3389/fncel.2020.588445 (PMC7705537; doi:10.3389/fncel.2020.588445)
Supplement: Supplementary file 1 [file Data_Sheet_1.PDF]

Supplementary Material

1 SUPPLEMENTARY FIGURES FOR THE TRACKING QUALITY METRIC

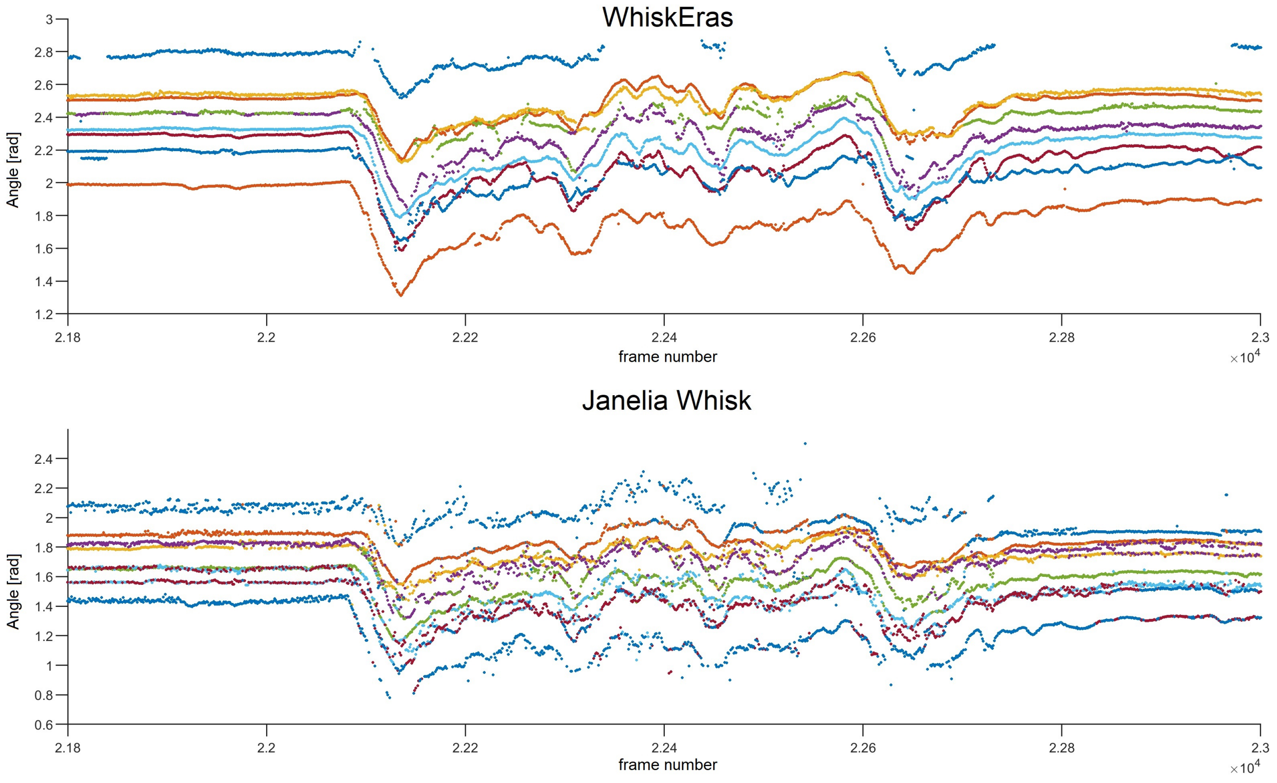

Figure S1. Video fragment A, frames 21800 to 23000, as tracked by WhiskEras and Janelia Whisk.

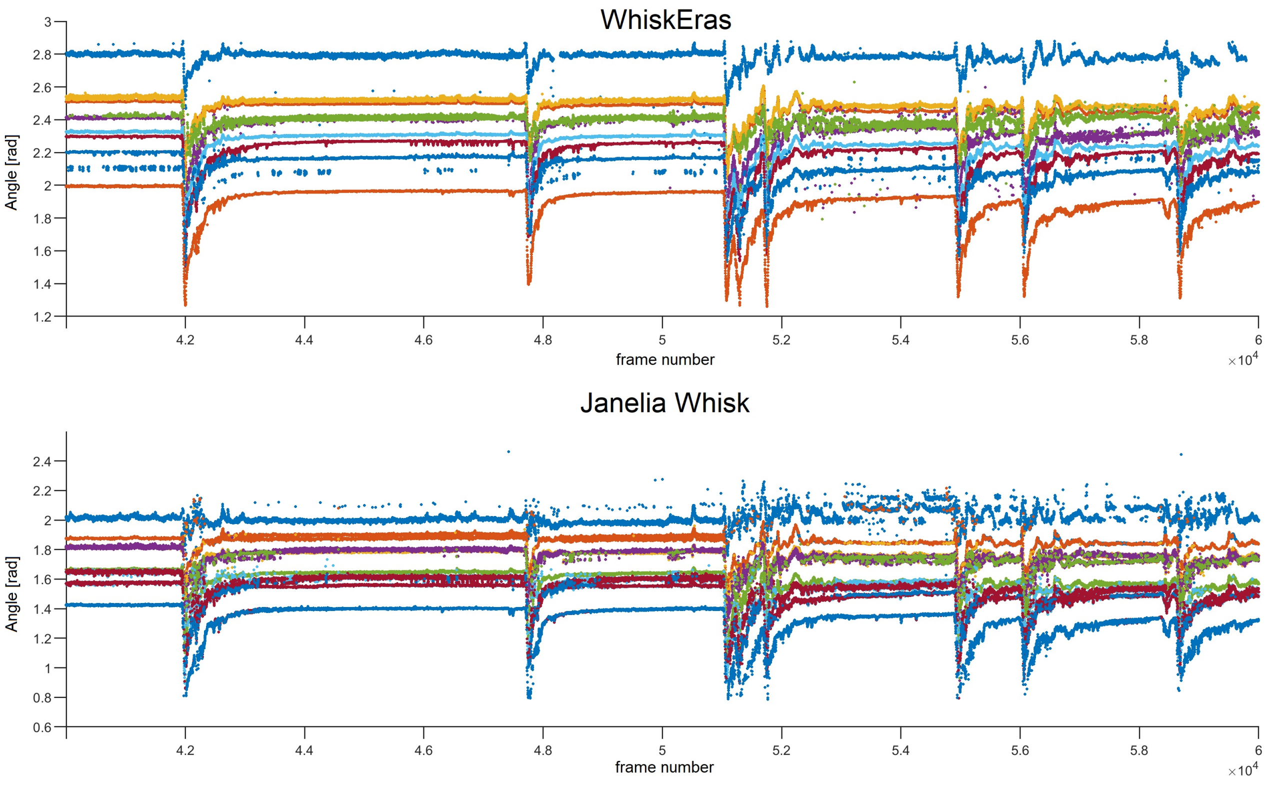

Figure S2. Video fragment A, frames 40000 to 60000, as tracked by WhiskEras and Janelia Whisk.

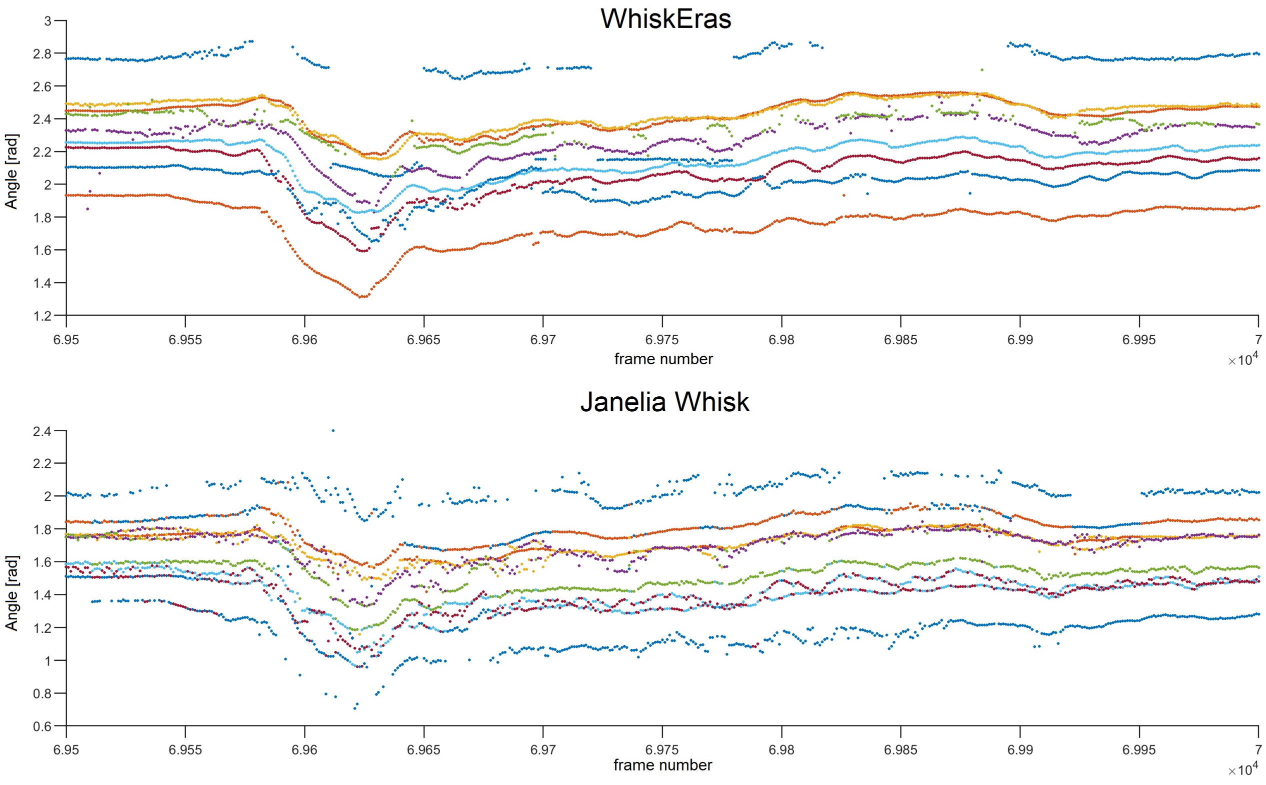

Figure S3. Video fragment A, frames 69500 to 70000, as tracked by WhiskEras and Janelia Whisk.

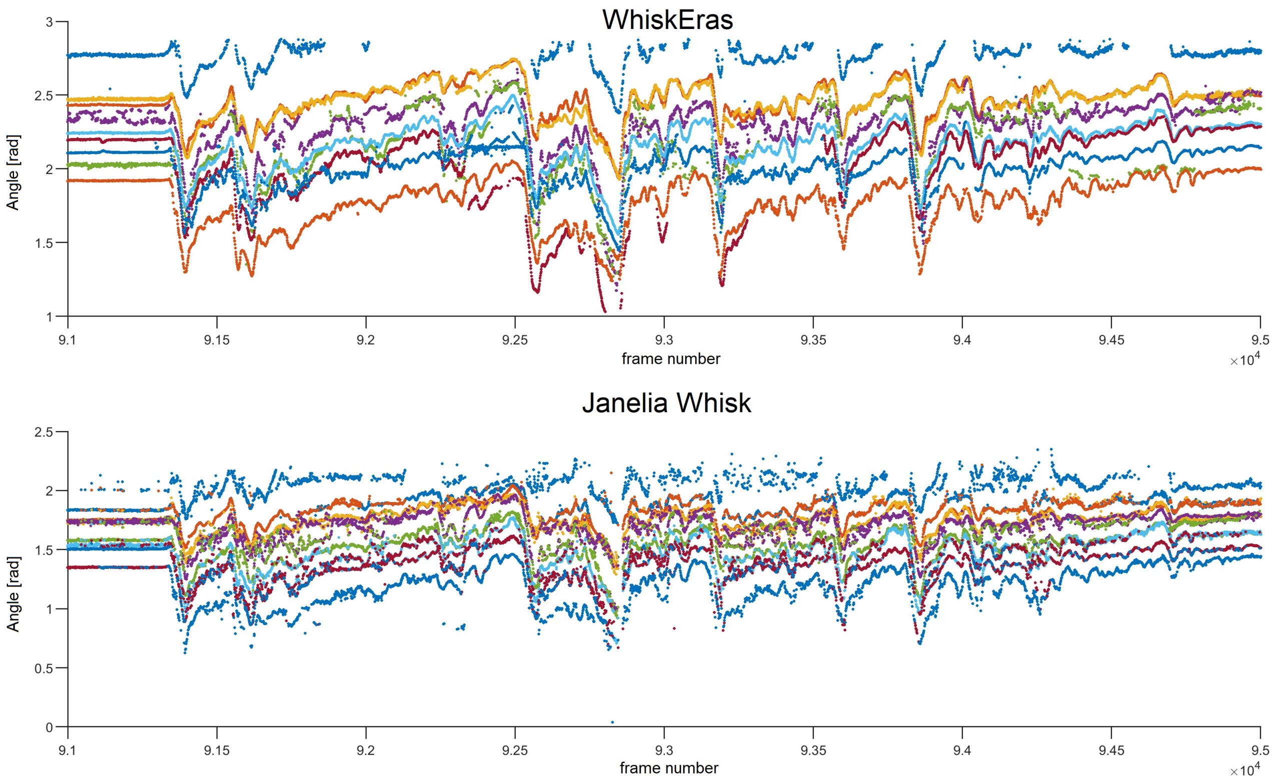

Figure S4. Video fragment A, frames 91000 to 95000, as tracked by WhiskEras and Janelia Whisk.

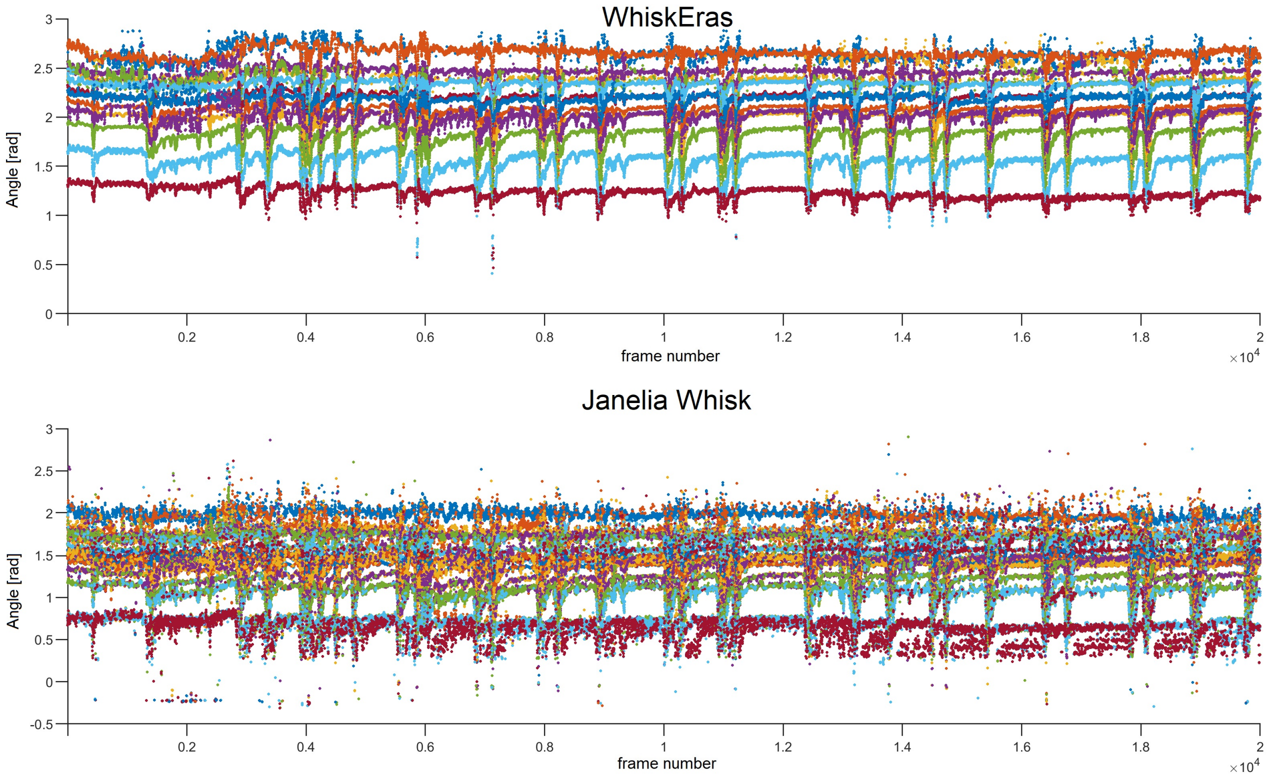

Figure S5. Video fragment B, frames 1 to 20000, as tracked by WhiskEras and Janelia Whisk.

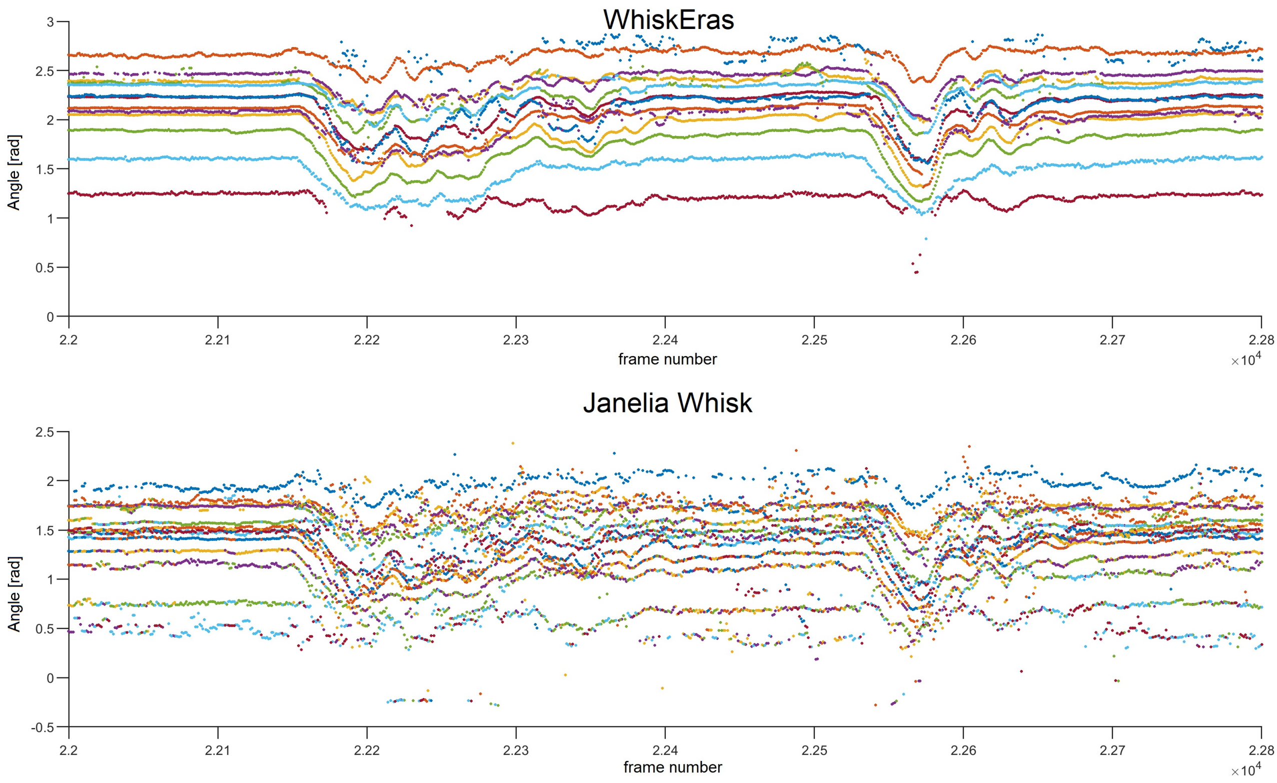

Figure S6. Video fragment B, frames 22000 to 22800, as tracked by WhiskEras and Janelia Whisk.

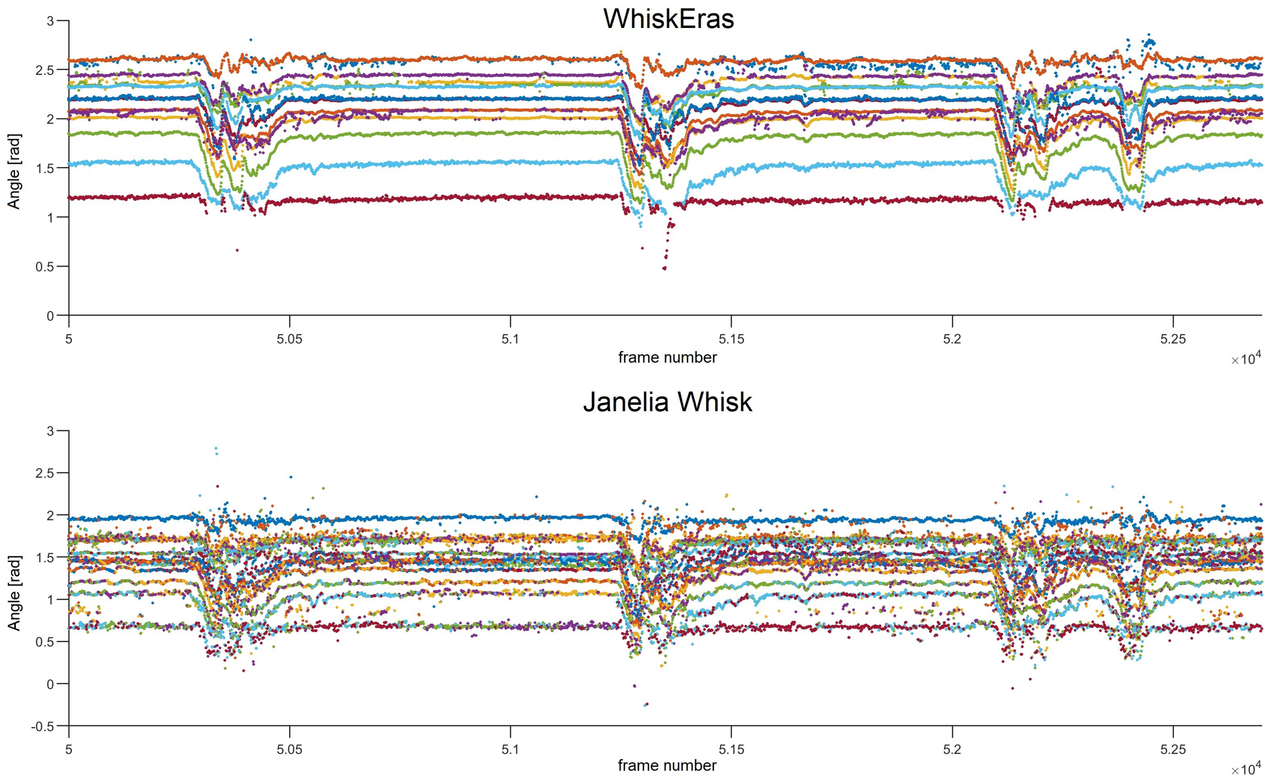

Figure S7. Video fragment B, frames 50000 to 52700, as tracked by WhiskEras and Janelia Whisk.

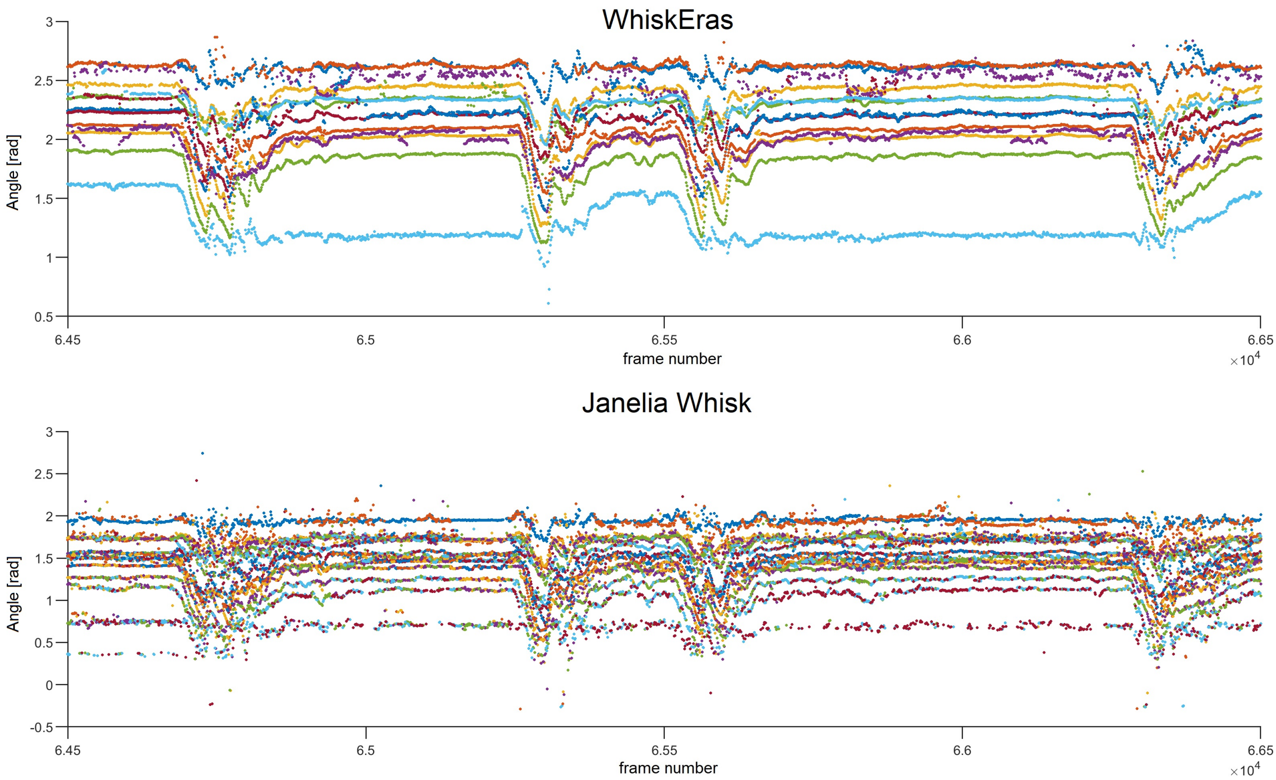

Figure S8. Video fragment B, frames 64500 to 66500, as tracked by WhiskEras and Janelia Whisk.

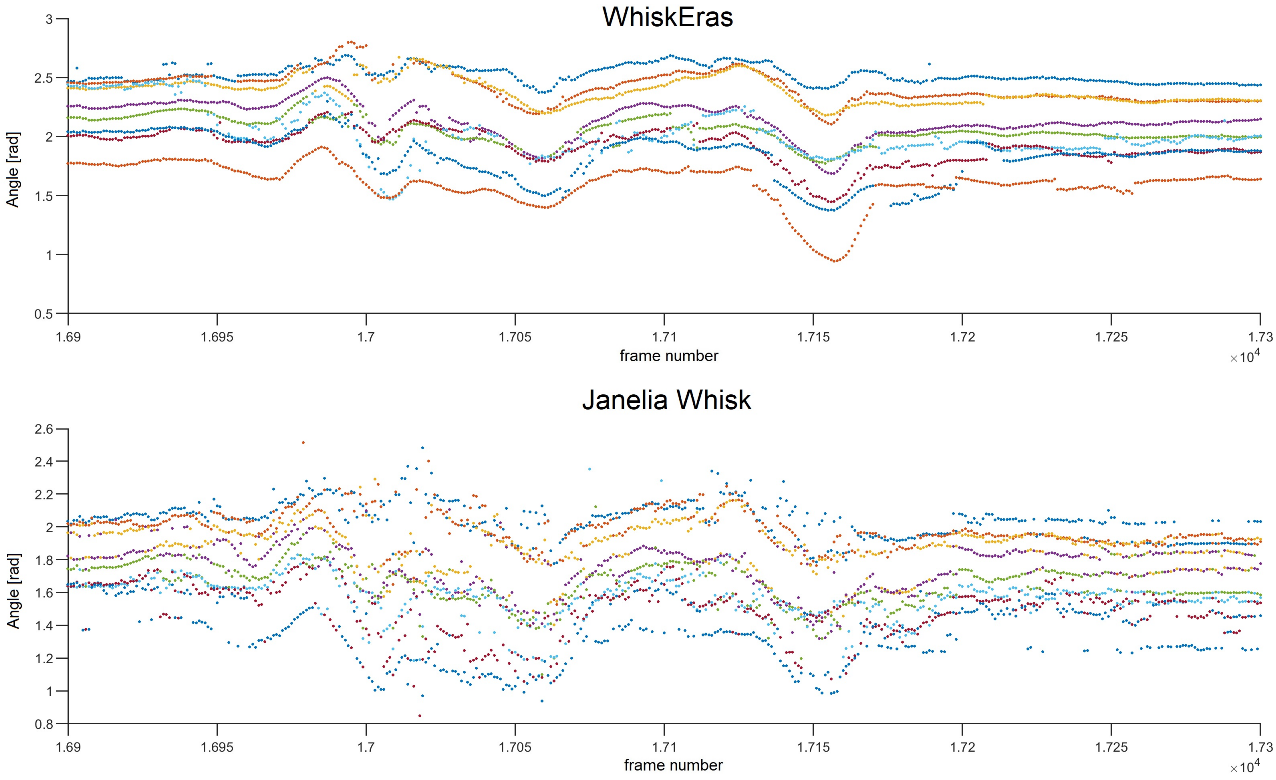

Figure S9. Video fragment C, frames 16900 to 17300, as tracked by WhiskEras and Janelia Whisk.

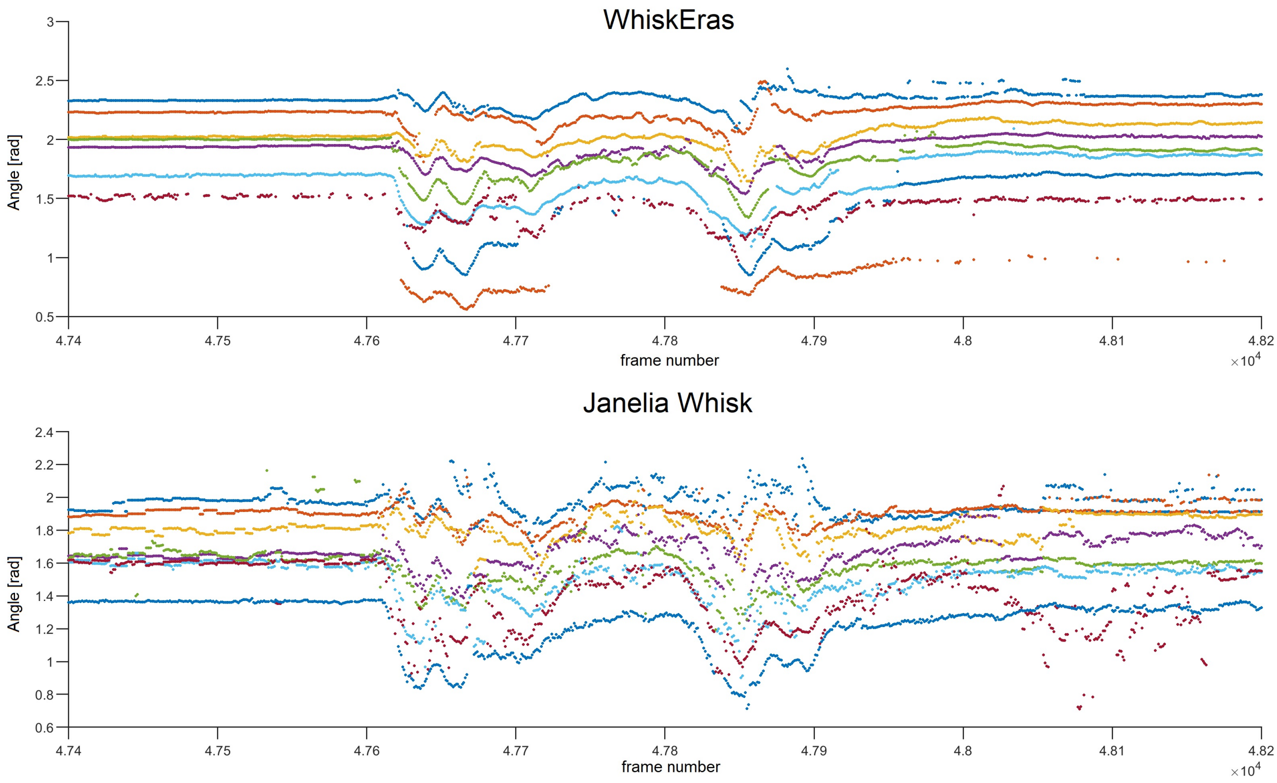

Figure S10. Video fragment C, frames 47400 to 48200, as tracked by WhiskEras and Janelia Whisk.

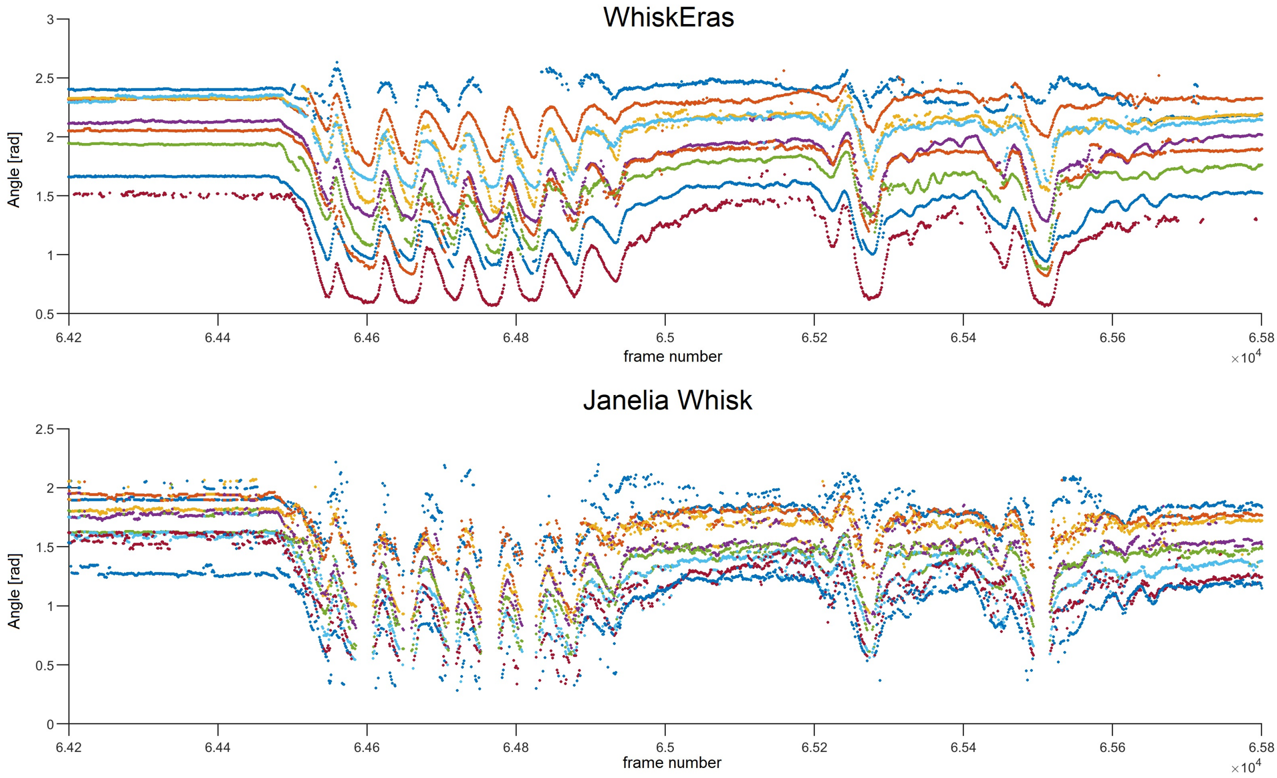

Figure S11. Video fragment C, frames 64200 to 65800, as tracked by WhiskEras and Janelia Whisk.

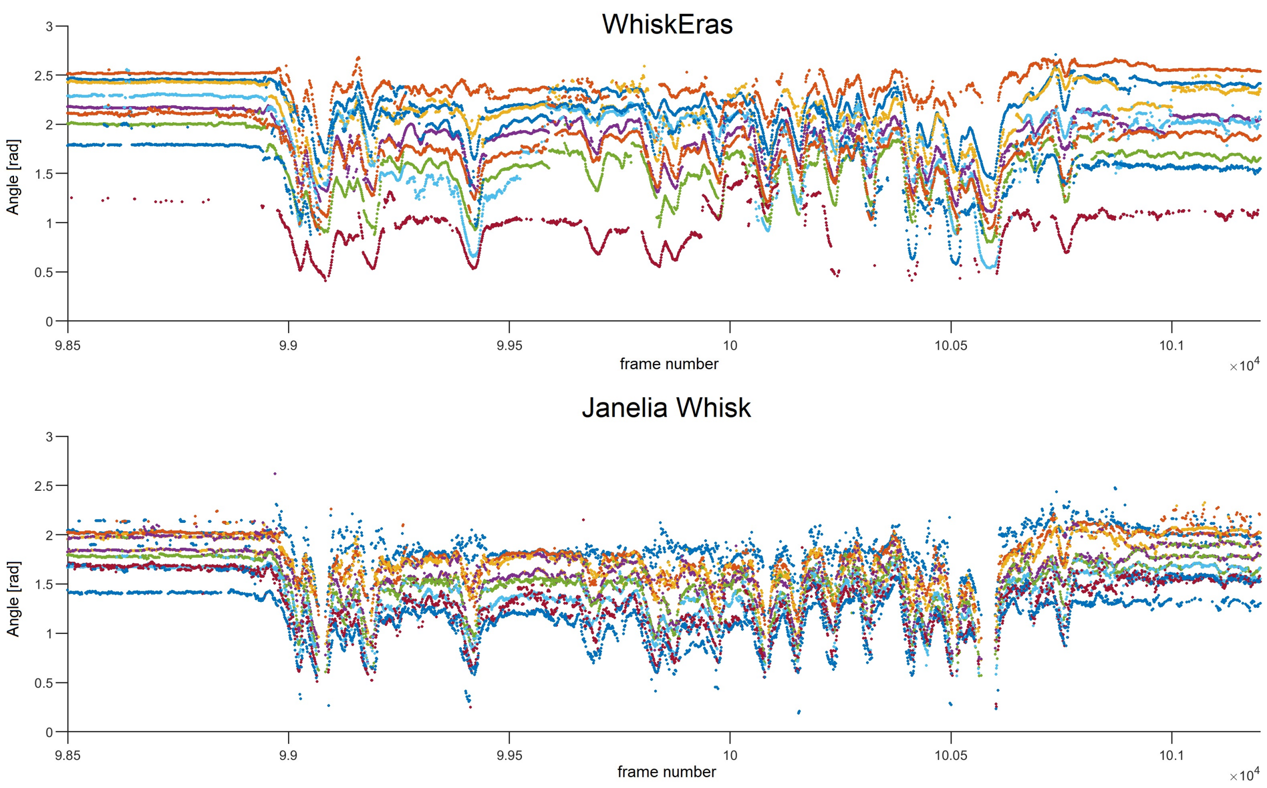

Figure S12. Video fragment C, frames 98500 to 101200, as tracked by WhiskEras and Janelia Whisk.

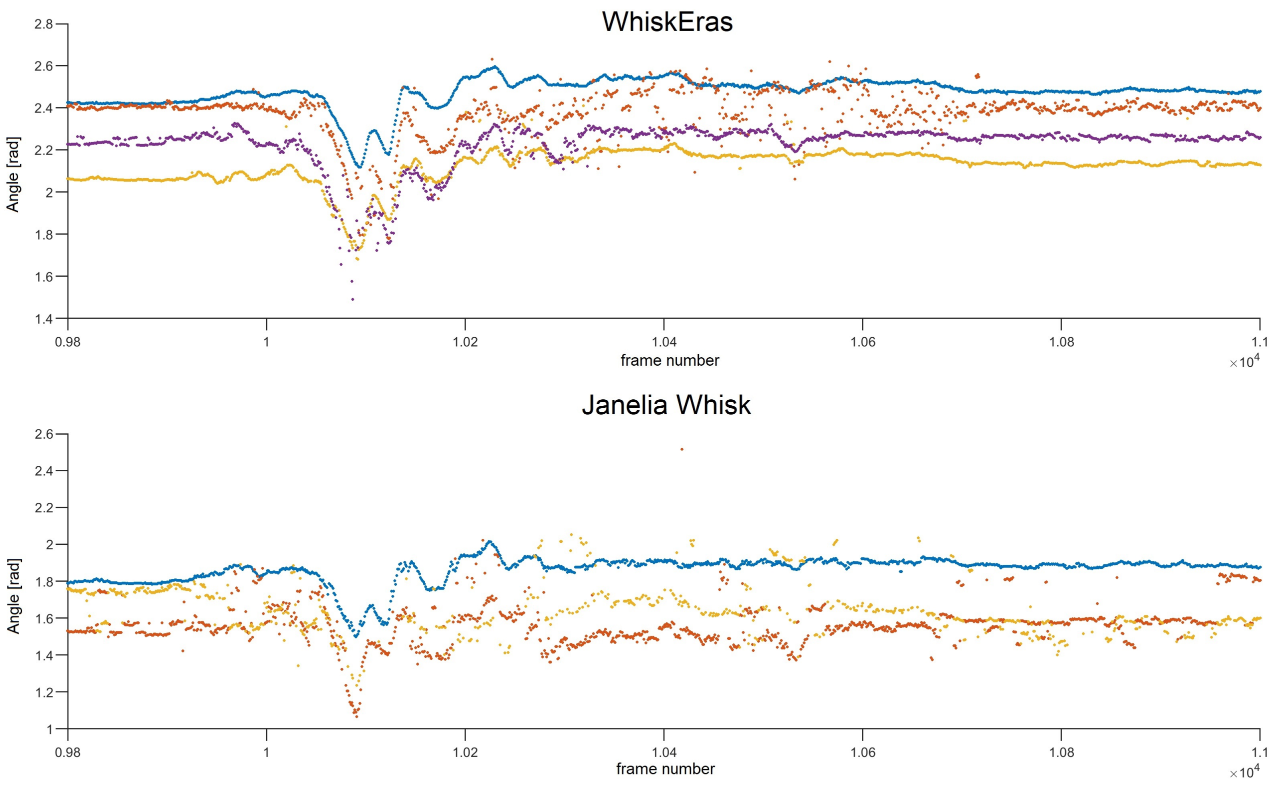

Figure S13. Video fragment D, frames 9800 to 11000, as tracked by WhiskEras and Janelia Whisk.

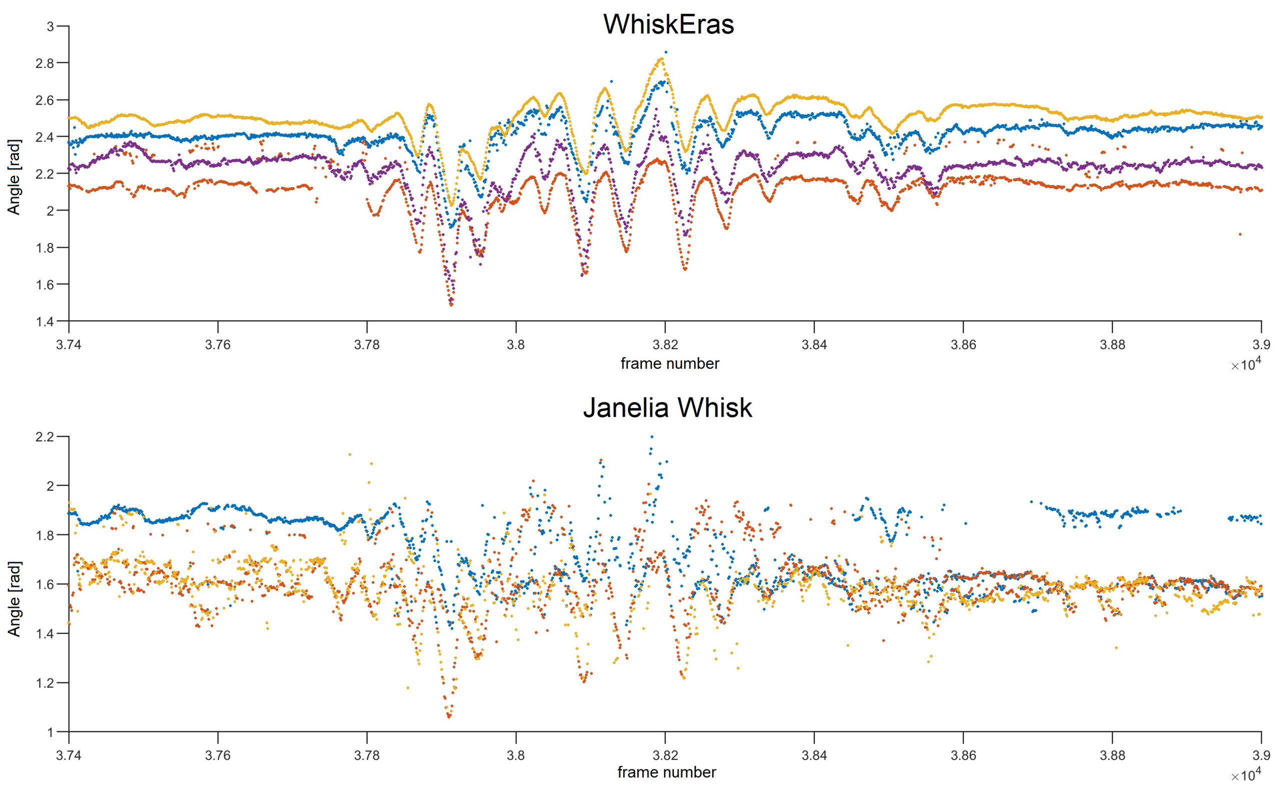

Figure S14. Video fragment D, frames 37400 to 39000, as tracked by WhiskEras and Janelia Whisk.

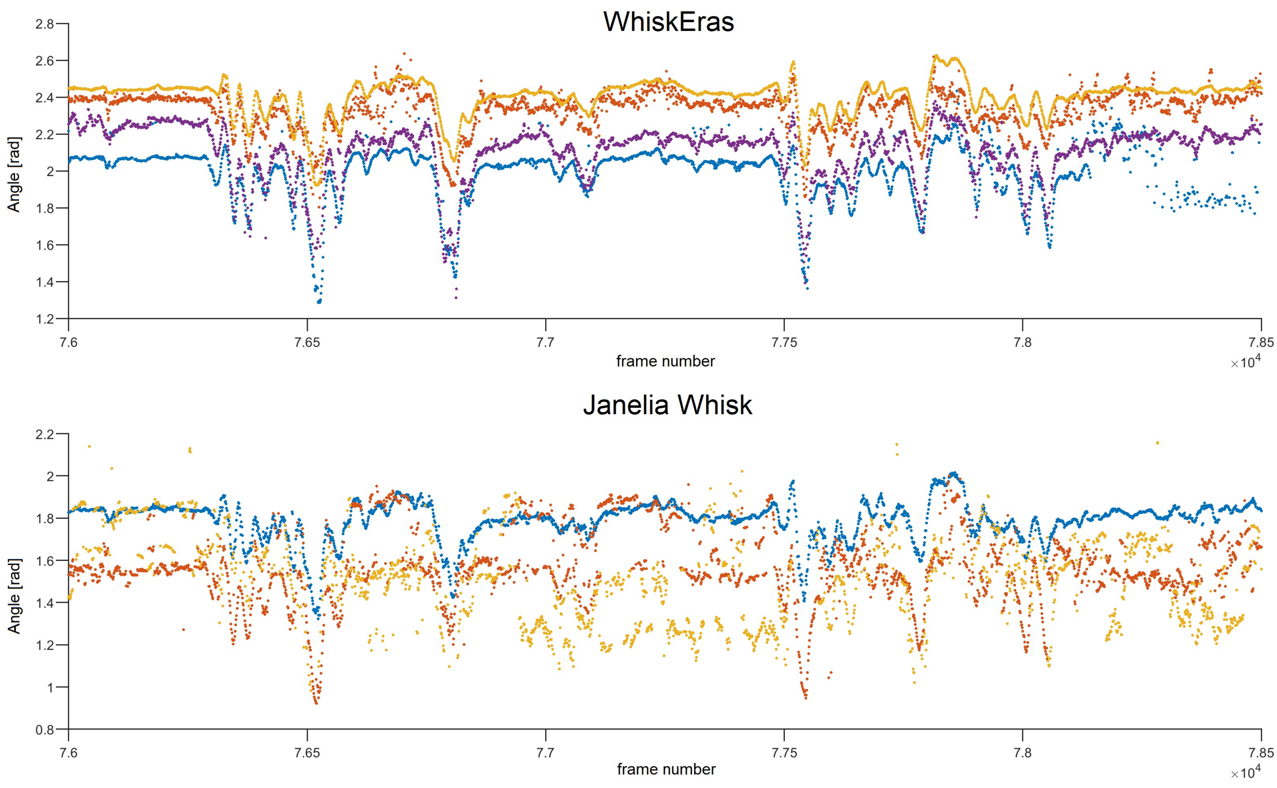

Figure S15. Video fragment D, frames 76000 to 78500, as tracked by WhiskEras and Janelia Whisk.

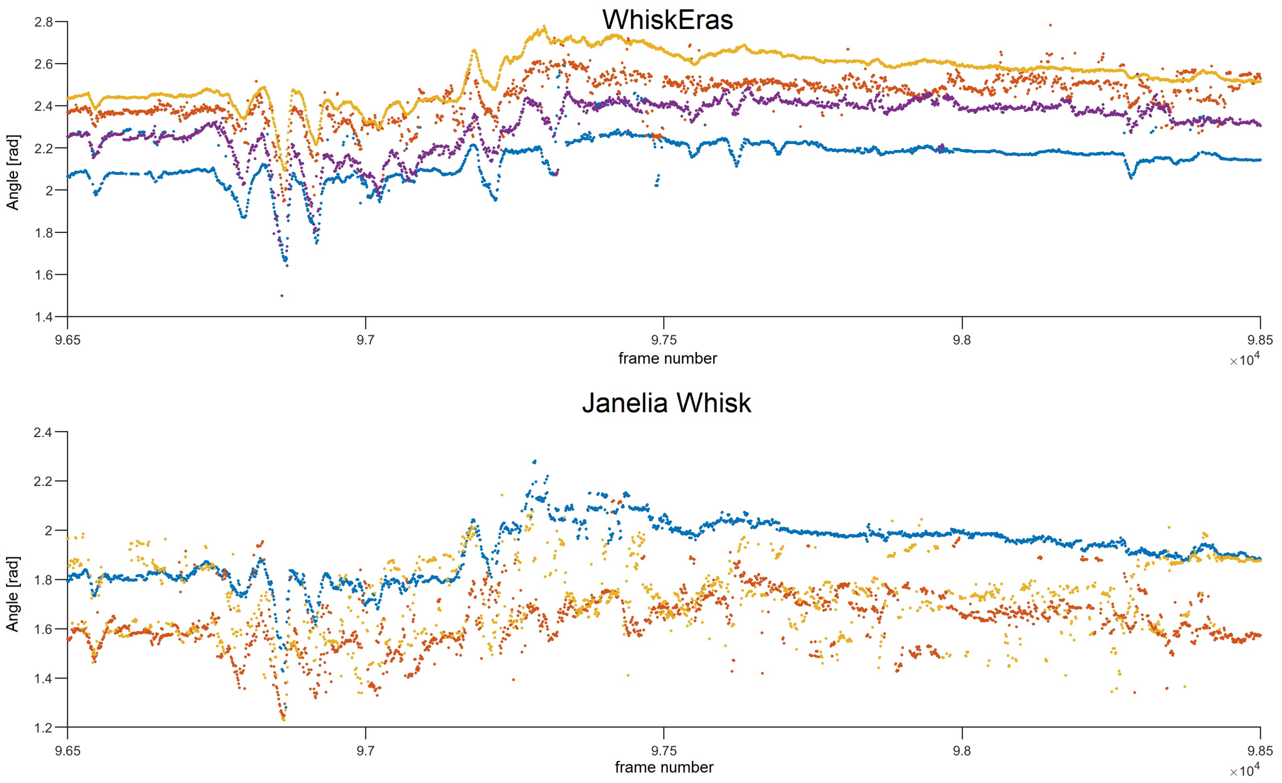

Figure S16. Video fragment D, frames 96500 to 98500, as tracked by WhiskEras and Janelia Whisk.
